# FcγR-driven chimeric receptor T cells in cancer therapy: a novel frontier in antibody-guided immunotherapy

**DOI:** 10.1186/s12935-026-04258-7

**Published:** 2026-04-16

**Authors:** Hany E. Marei, Giacomo Pozzoli, Sara Caratelli, Carlo Cenciarelli

**Affiliations:** 1 Department of Cytology and Histology, Faculty of Veterinary Medicine, Mansoura University, Mansoura, 35116 Egypt; 2 Institute of Pharmacology, Università Cattolica del Sacro Cuore, Rome, Italy; 3 Pharmacology Unit, Fondazione Policlinico Universitario A. Gemelli IRCCS, Rome, Italy; 4 Institute of Translational Pharmacology (IFT), National Research Council (CNR), Rome, Italy

**Keywords:** Solid tumors, Adoptive cellular therapy (ACT), Fc gamma receptors (FcγRs), CD16a (FcγRIIIa), CD32a (FcγRIIa), CD64a (FcγRI), Chimeric receptor (CR) T cells, Antibody-dependent cellular cytotoxicity (ADCC), Monoclonal antibody-based immunotherapy, Engineered T cells, Immunotherapy for solid tumors, FcγR-CRT cells therapy, Tumor microenvironment (TME), Synthetic immunoreceptors, Gene editing for immunotherapy, Checkpoint inhibitors, Personalized cancer therapy, CRISPR/Cas9 in cell therapy, Clinical translation of engineered T cells

## Abstract

This review article looks at a novel and flexible framework in adaptive cell treatment that enhances tumor targeting using Fc gamma receptor (FcγR)-based chimeric receptors. Though chimeric antigen receptor (CAR) T cell therapies have shown notable effectiveness in hematologic malignancies, their efficiency against solid tumors is limited by tumor heterogeneity, immunosuppressive tumor microenvironments (TMEs), and off-target toxicities. By replacing the conventional scFv domain with the extracellular domains of FcγRs—including CD16a, CD32a, or CD64a—FcγR-CR T cells provide a unique method allowing T cells to be steered by monoclonal antibodies (mAbs) targeting several tumor-associated antigens (TAAs). Emphasizing their advantages in flexibility, reversibility, and compatibility with present antibody therapy, the review clarifies the mechanisms of action, preclinical developments, and clinical promise of FcγR-CR T cells. Future directions include increasing specificity by means of affinity-engineered FcγRs and Fc-modified monoclonal antibodies, generating universal allogeneic FcγR-CR T cells for immediate use, using regulatable expression systems to improve safety, and combining these engineered cells with immune checkpoint inhibitors or metabolic modulators to overcome tumor microenvironment resistance. Overall, FcγR-CR T cells offer a hopeful next-generation immunotherapeutic approach able to handle the current CAR T cell constraints and change precision oncology, particularly for aggressive and treatment-resistant solid cancers.

## Introduction

While chimeric antigen receptor (CAR) T-cell therapies exhibit tremendous potential for treating hematological cancers, they have not demonstrated equivalent effectiveness against solid tumors. Solid tumors (including those found in the brain, breast, lung, prostate and colon) represent a large proportion of cancer deaths globally and are typically aggressive, resistant to treatment and offer a bleak prognosis despite advances in surgery, radiation and systemic therapy [1]. Significant challenges to providing an effective immunological treatment for solid cancers due to the high levels of intratumor heterogeneity, immune resistance, and an extremely immunosuppressive environment create obstacles for producing sustained immune responses against the tumors long-term [2, 3].

The development of CAR-T cell immunotherapy has provided an innovative means to treat solid tumors through genetic redirection of T cells toward tumor-associated antigens (TAAs) [4, 5]. Experience gained through the successful application of CD19-targeted CAR-T cells for hematological cancers resulted in several FDA approvals based on evidence of the strategy’s effectiveness [6]. A typical CAR consists of the following: an extracellular single-chain variable fragment (scFv), along with intracellular signaling and costimulatory domains that recognize an antigen and activate a T cell [7]. Although CAR-T cell therapy has been introduced for the treatment of hematological malignancies, it is still limited in its application for solid tumors due to the following limitations: (1) Limited availability of the antigens that can be targeted on solid tumors. (2) On-target, off-tumor toxicity. (3) Cytokine release syndrome (CRS) (4) An increased likelihood of developing dysfunction early in T-cell development due to prolonged (chronic) exposure to Herceptin (Herceptin-driven), as well as inhibiting inflammation signals from the Microenvironment of the tumor [8–10]. In addition, the technical complexity and resource demands related to manufacturing CAR-T cells at a clinical grade for clinical use are so great that currently, they are not completely feasible for large-scale clinical use [11].

A number of these limitations relate to the use of scFv-based TAA recognition. Because no TAA is universally expressed, it is necessary to develop scFvs that are specific for all different TAAs, which leads to a lack of flexibility, an increase in the risk of antigen escape, and less scalability [12]. In addition, the inability of conventional CAR-T cells to adapt dynamically to evolving antigen landscapes may create an opportunity for the emergence and outgrowth of antigen-negative tumor variants.

One way to address the obstacles we face in cell therapy is to use chimeric antigen receptor (CAR) T cells that incorporate the Fc portion of antibodies (also called Fcγ). The idea behind this approach is to leverage the biological activity of the Fc portion of antibodies to enhance the engineered T cell’s ability to kill cancer cells bound by an antibody, using currently available monoclonal antibodies. By adding the extracellular portion of CD16a (FcγRIIIa) to the CAR construct, the engineered T-cell will be able to recognize and kill antibody-bound tumor cells in conjunction with currently used monoclonal antibodies. ADCC is a well-established immune mechanism that CD16a-CR T cells utilize to kill tumor cells. The modularity of this design allows the use of one or more therapeutic antibodies to target multiple tumor antigens, as well as to control T-cell function through administration at high or low doses or by stopping treatment altogether. These concepts have been examined in preclinical models, demonstrating that CD16a-CR T cells or NK cells can effectively inhibit tumor growth when combined with therapeutic monoclonal antibodies in vivo [13–22]. Early clinical trial results (NCT03189836, NCT02776813, NCT03266692) are being evaluated to determine the safety and feasibility of this combination of therapies.

Other Fcγ receptors, such as CD32a (FcγRIIa) and CD64a (FcγRI), in addition to CD16a, have been studied as potential components of chimeric receptors due to their differing affinities and functions. The Fcγ receptor family serves as a bridge connecting the innate and adaptive immune systems by enhancing phagocytosis and ADCC. As a result, some Fcγ-Rs may be suitable candidates for the development of engineered therapeutics for patients living with solid tumors in an immunosuppressive local tumor microenvironment [27, 28].

This review provides an in-depth examination of the use of only CD16a-, CD32a-, and CD64a- based FcγR-CR T cells in solid tumor treatment. This includes a focus on the biological basis of these cells, their engineering methods, their preclinical advances, and the obstacles to moving them into clinical practice. The evidence presented demonstrates that monoclonal antibody therapy continues to impact and broaden the scope of FcγR-based cellular immunotherapy, enabling the development of precision immuno-oncology and the potential to overcome the existing limitations of CAR-T cell therapies, creating an even greater opportunity to treat solid malignancies more effectively than ever before.

## FC gamma receptors (FcγR) and their role in immunotherapy

**Fig. 1:**
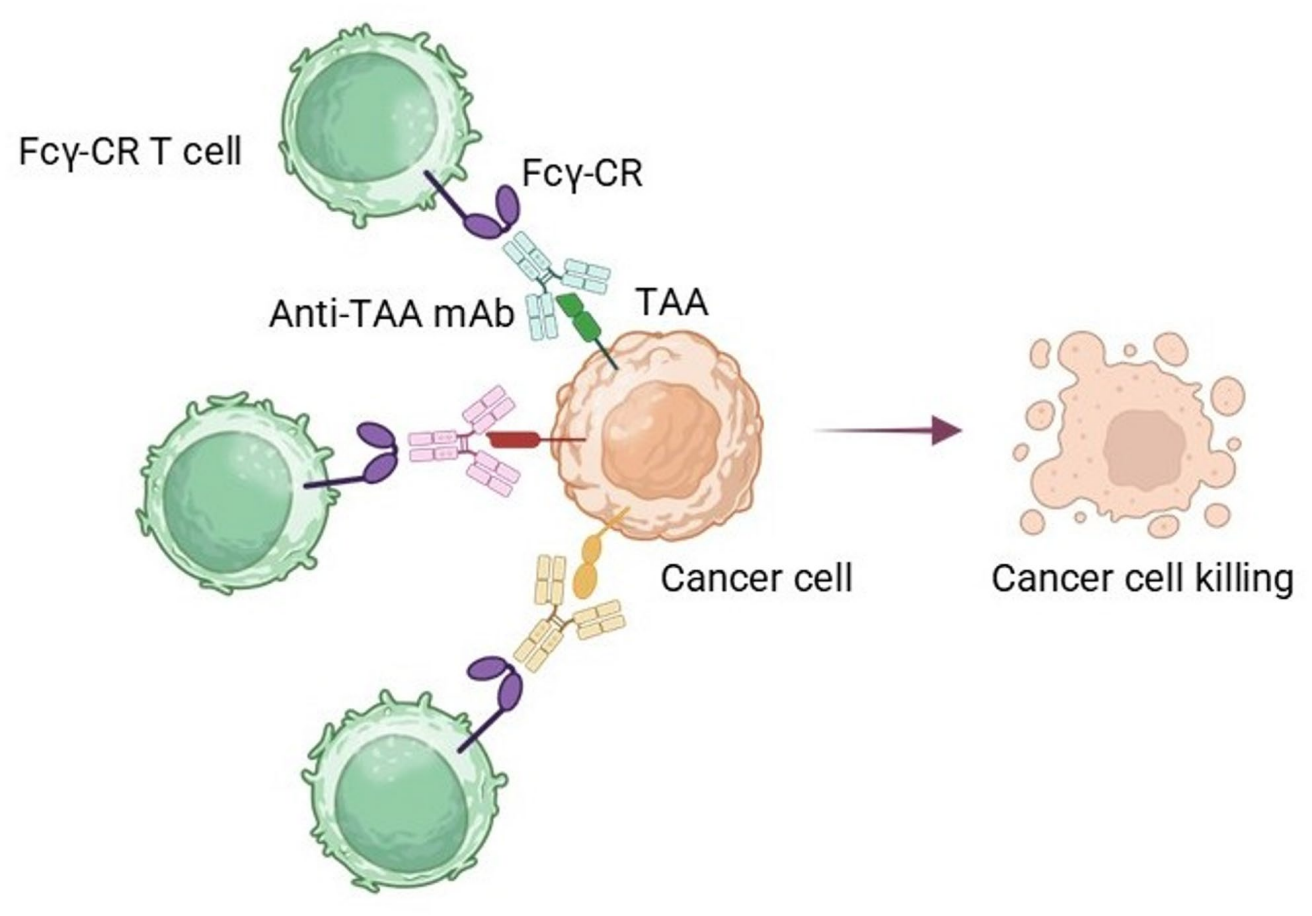
Fcγ receptor–driven chimeric receptor T-cell–mediated tumor cell cytotoxicity. The graphical representation shows how Fcγ chimeric receptors expressed on engineered T cells (Fcγ-CR T cells) destroy tumor cells by recognizing tumor-associated antigens (TAAs) with antibodies. Specifically, the Fcγ-CRs on the T cells recognize the Fc portion of the mAbs that, in turn, bind to the tumor cell’s surface. This leads to the formation of an immune synapse between the Fcγ-CR T cell and the TAA on the tumor cell surface, the T cell’s activation, and subsequent coordinated cytotoxic killing of the tumor cell. The use of anti-TAA mAbs in this way provides a method for effectively killing cancer cells while still allowing flexibility in targeting with different therapeutic antibodi

Fundamental in immune control and effector responses, FcγRs are a group of immunoglobulin G (IgG)-binding proteins. Mainly located on different immune cells—including NK cells, macrophages, neutrophils, dendritic cells, and specific T cell subsets—these receptors connect the adaptive and innate immune systems by binding to the Fc region of IgG antibodies (Fig. 1), hence enabling vital functions such as phagocytosis, antibody-dependent cellular cytotoxicity (ADCC), cytokine release, and immunological complex clearance [23]. Figure 1 shows the proper orientation of the antibody, which corresponds to the way the interaction between the antibody and its target actually occurs physiologically. Antibody orientations that are inverted or are not functional do not reflect the typical physiological behavior of an antibody and its corresponding receptor(s). In an antibody-driven system, engineered T cells (CR T cells) express T-cell receptors and bind to proteins on the surface of target cells (e.g., cancer cells) that express tumor-associated antigens. The Fc portion of the antibody will be available to interact with the Fc receptors expressed by the engineered T cells. Therefore, when binding occurs, the spatial arrangement of the antibodies enables cross-linking and clustering of FcγR molecules at the immunological synapse, which is necessary to initiate signaling through the intracellular subunit CD3ζ and costimulatory motifs. Therefore, although the T-cell receptor does recognize the antigen, the primary driving force behind T-cell activation in this context is the interaction of the Fc region of the antibody with the corresponding FcγR on the engineered T cell, similar to the classic natural killer cell-mediated antibody-dependent cellular cytotoxicity or ADCC function. By using this antibody orientation to explain how T cells translate the information obtained from antibodies into T-cell activation, we provide direct evidence of the physiological implications of this antibody orientation for T-cell function.

Recent advances in understanding Fcγ receptor (FcγR) biology have shown that FcγRs play a wide range of roles, not just in mediating the classical antibody-dependent effector functions, but also in regulating both humoral and cellular immune responses, bridging the innate and adaptive immune systems. The interaction of FcγRs on myeloid cells and lymphoid subsets enables the execution of many different antibody-induced activities, including phagocytosis, clearance of immune complexes, modulation of antigen presentation, and cytokine production [24]. Additionally, the engagement of FcγR has been proposed to be a key factor in mediating the efficacy and mechanism of action of immune checkpoint antibodies in cancer immunotherapy, whereby FcγR engagement varies based upon the IgG isotype type or engineered Fc variant, with distinct effects on both receptor binding, effector recruitment and antitumor activity, whereby the impact of FcγR interactions can determine the clinical outcomes associated with the depletion of regulatory T cells and the enhancement of effector function [25, 26]. Furthermore, based on the aforementioned information and hypothesis, it has been proposed that FcγRs function as “antibody checkpoints” that regulate antitumor immunity through mechanisms analogous in concept to T-cell inhibitory checkpoints but functionally distinct. Therefore, the different outcomes caused by the engagement of an Fc with a specific target and design will be both beneficial and detrimental [27, 28]. These reviews promote the need to account for the biology of FcγRs in the design and interpretation of antibody-based therapies, especially in the development of next-generation immune modulators.

**Fig. 2:**
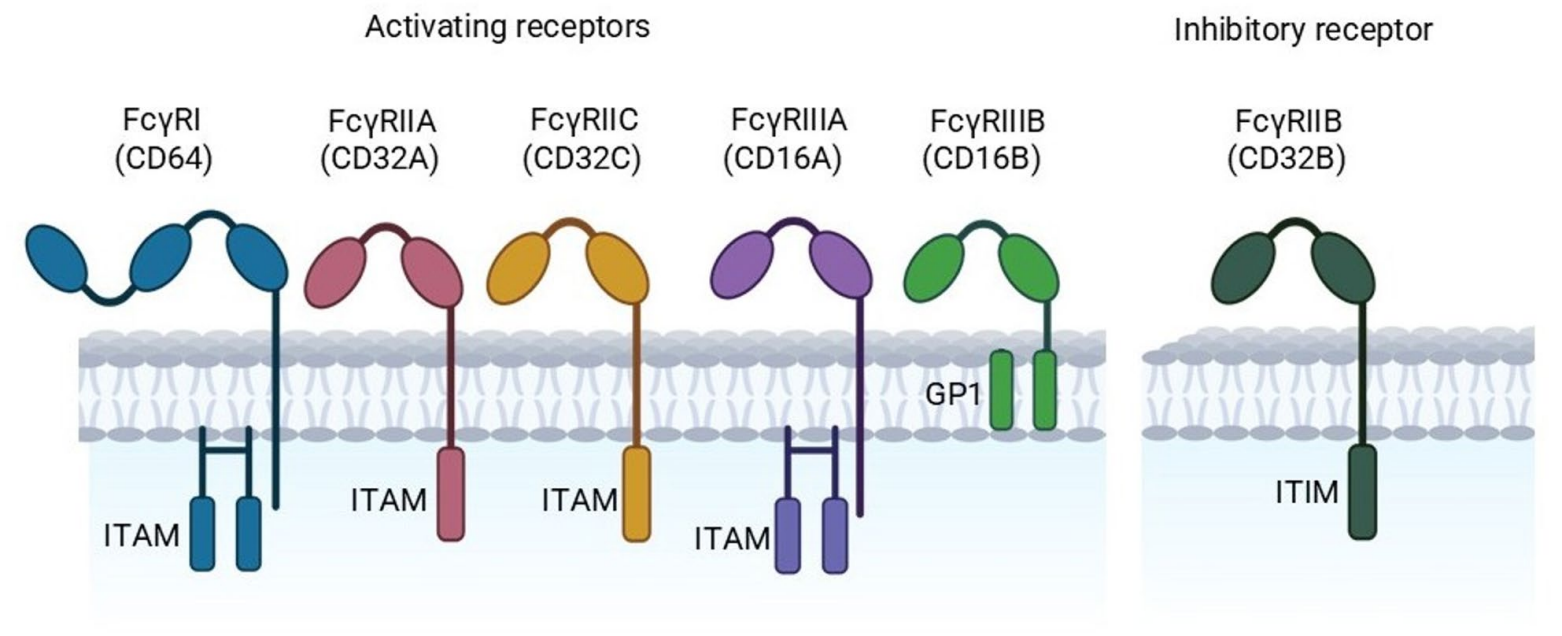
Structure and signaling properties of human Fcγ receptors. There are three primary categories of Fc (Ig) Receptor types depending on their mechanisms of subcellular signaling; these are divided into two types: ‘Activating’ Receptors or ‘Inhibitory’ Receptors. ‘Activators’ such as FcγRI (CD64), FcγRIIA (CD32A), FcγRIIC (CD32C), FcγRIIIA (CD16A), and FcγRIIIB (CD16B) are categorized in part by the Immunoreceptor Tyrosine-Based Activation Motifs (ITAMS). Activating Receptors exhibit multiple signaling pathways through interactions with accessory molecules or ligands, e.g., FcγRIIIB (CD16B) is glycosylphosphatidylinositol (GPI)-anchored; FcγRI (CD64) and FcγRIIIA (CD16A) form dimers, and signals are transmitted indirectly through bound membrane receptors. Whereas inhibitory receptors, such as FcγRIIB (CD32B), can impede activation via an Immunoreceptor Tyrosine-based inhibition Motif (ITIM). If one considers that antibody function is ‘dependent’ on the balance between activating/inhibitory receptors signaling, then it is evident that the receptors’ signaling results in a broad spectrum of antibody effects/activities

According to their structure and function, human FcγRs are classified into three main types: FcγRI (CD64), FcγRII (CD32), and FcγRIII (CD16). Mostly present on monocytes, macrophages, and dendritic cells, FcγRI (CD64) is a high-affinity receptor that binds monomeric IgG1 and IgG3 with notable affinity. Comprising both activating receptors—FcγRIIa and FcγRIIc—FcγRII (CD32) **(**Fig. 2**)** also includes the only inhibitory Fcγ receptor, FcγRIIb, which is crucial for maintaining immunological tolerance and preventing too strong immune activation [29]. Comprising two isoforms, FcγRIII (CD16) includes FcγRIIIa (CD16a), found on NK cells, certain T cells, and macrophage subgroups, and FcγRIIIb (CD16b), expressed only on neutrophils. Both isoforms are low-affinity IgG receptors that help to identify and remove immune complexes [22, 23, 30–37].

Its ability to enable potent ADCC has made FcγRIIIa (CD16a) a key receptor in immunotherapy. Activation of CD16a on NK cells causes the lysis of antibody-coated target cells, a mechanism used in mAbs therapies for cancer and viral infections [38]. Furthermore, CD16 has been widely used in CR T cell engineering, in which T cells are genetically modified to express FcγRs, allowing therapeutic antibodies to redirect them to destroy cancer cells. For cancer immunotherapy, this approach provides a flexible and adaptable structure (34,36,39,40).

Essential components of the immune system, FcγRs mediate the link between antibody recognition and cellular immune responses. Their discovery has tremendously helped us understand the immune system, particularly in the realm of immunotherapy. The first studies on antibody interactions with immune cells identified these receptors, which have since been thoroughly characterized for their roles in immunological modulation [41]. FcγRs are predominantly expressed on innate immune cells, including NK cells, macrophages, neutrophils, and dendritic cells, highlighting their important role in natural immunity [42]. By detecting IgG-opsonized targets, FcγRs link humoral and cellular defense, enabling effector cells to discover and destroy aberrant or infectious cells [43]. Essential for host defense, this role significantly affects the efficacy of treatments for cancer and infectious diseases.

Three central activating receptors among the Fcγ receptors—CD16a, CD32a, and CD64a—show distinct expression patterns across various immune cells, enabling distinct immunological activities. Mostly found on natural killer (NK) cells, monocytes, and macrophages, CD16a (FcγRIIIa) facilitates ADCC and phagocytosis. CD16a plays an important role in tumor immunosurveillance by engaging IgG-opsonized targets, inducing effector cells to mount deadly responses [44]. On macrophages, neutrophils, and dendritic cells, CD32a (FcγRIIa) is found and helps clear immune complexes. By facilitating the absorption and breakdown of immune complexes, CD32a increases antigen processing and presentation, thereby boosting adaptive immune responses [45]. Mostly expressed on monocytes and macrophages, CD64a (FcγRI) binds IgG with High Affinity. This strong link increases phagocytic activity, helping eliminate infections and cancerous cells efficiently. The strong immune activation induced by CD64a underscores its potential as an immunotherapy target [46].

NK cells are key players in immune surveillance and tumor eradication, a subset of innate lymphoid cells (ILCs). Highly expressed on NK cells, FcγRIIIa (CD16a) drives ADCC. On target cells, FcγRIIIa binds the Fc region of IgG antibodies, triggering NK cell activation and the release of cytotoxic granules, thereby destroying the target cell. This approach demonstrates that FcγRs directly eliminate antibody-coated targets, a process necessary for controlling infections and tumor development. FcγRs’ action mechanism involves their ability to bind the Fc region of IgG antibodies, generating immune complexes that trigger different cellular responses [47]. FcγRs undergo conformational changes upon activation that trigger intracellular signaling pathways, leading to phagocytosis, cytokine production, and cytotoxicity, among other consequences. The type of FcγR activated and the cellular environment determine the specific response, enabling tailored immune responses to different stimuli [48].

## Design, engineering, and mechanism of action of FcγR-CR T cells

The innate immune system is the body’s first line of defense against infections, virally infected cells, and neoplastic cells. This includes diverse myeloid and lymphoid cells, such as granulocytes, monocytes/macrophages, and dendritic cells (DCs) [49, 50]. Cytokine production [51, 52], phagocytosis [50], cytotoxic actions [53], and tissue repair facilitation following injury [54–56], help myeloid cells regulate inflammatory responses. Tissue homeostasis and mucosal immunity are significantly influenced by lymphoid subsets, including natural killer (NK) and innate lymphoid cells (ILCs). Central to these functions are Fc gamma receptors (FcγRs), which control the activity of NK cells, ILCs, dendritic cells, and other myeloid cells [57].

The choice of CD16a (FcγRIIIa) as the primary Fcγ receptor for creating chimeric receptors on T cells arose from its major contribution to antibody-dependent cellular cytotoxicity (ADCC). This same mechanism also explains how therapeutic monoclonal antibodies (a form of treatment) work [58, 59]. The CD16a molecule interacts with the IgG1 or IgG3 antibody that has been coated on a cancer cell (target cell) to activate (induce) cell death, as shown in Fig. 1. This activation will occur through several signal pathways, including ITAMs [60], and by activating other pathways, such as tumor necrosis factor (TNF)-α. Clinical trial data have shown that high-affinity CD16a variants, specifically the valine variant at position 158, exhibit stronger IgG binding to CD16a and therefore greater clinical responses to therapeutic monoclonal antibodies such as Rituxan, Herceptin, and Erbitux [61, 62]. Based on these findings, CD16a has been used to develop engineered T-cell platforms for cancer therapy. Early studies supporting this concept provided proof-of-principle data showing that chimeric receptor T cells expressing CD16a, in the presence of antibody, could eliminate antibody-coated tumor cells without requiring MHC interaction [63]. Thus, the biological and clinical underpinnings of using CD16a to develop FcγR-driven chimeric receptor T-cell therapies are well established.

In controlling infections, inflammation, and tissue remodeling, FcγRs enable important immune processes, including antibody-dependent cellular cytotoxicity (ADCC), antibody-dependent phagocytosis (ADP), and cytokine release [64–69]. Apart from one inhibitory receptor, CD32B (FcγRIIB), there are three main types of activating Fcγ receptors: CD64 (FcγRI), CD32 (FcγRIIA/C), and CD16 (FcγRIIIA/B), as shown in Fig. 2. These receptors show varying affinity for immunoglobulin G (IgG) [67, 70–72]. While CD32 and CD16 exhibit low-affinity binding and are polymorphic, CD64 is a high-affinity receptor expressed on interferon-activated granulocytes, monocytes, macrophages, and dendritic cells [73, 74].

The capacity of engineered CD16A-expressing T cells to facilitate ADCC against B-cell malignancies was first established with rituximab [75], prompting the development of CD16A CR T cells aimed at solid tumors [14, 17, 76]. When paired with IgG2 mAbs, such as panitumumab, NK cells exhibit ADCC, whereas CD32-positive myeloid cells show a strong response [77]. Researchers developed CD32-CR T cells to tackle this problem. To do so, the scFv domain of standard CARs should be replaced with the extracellular domain of CD32. This approach allows CD32-CR T cells to bind to IgG2 antibodies even in the presence of serum and to be directed toward EGFR-overexpressing cancer cells using panitumumab [17, 21, 22, 78].

Apart from antibodies, FcγRs can engage with pentraxins—soluble proteins influencing innate immunity, such as C-reactive protein and serum amyloid P (SAP) [79, 80]. In the absence of antibodies, CD16A has been shown to bind nonspecific surface ligands on cancer cells, triggering NK cell cytotoxicity [81]. Although ligands for CD32 have not yet been identified, previous work demonstrates the presence of CD32-specific ligands on cancer cells using CD32-CR T cells as biosensors. These ligands, interacting with CD32-CR, trigger T-cell activation and cytotoxicity.

**Fig. 3:**
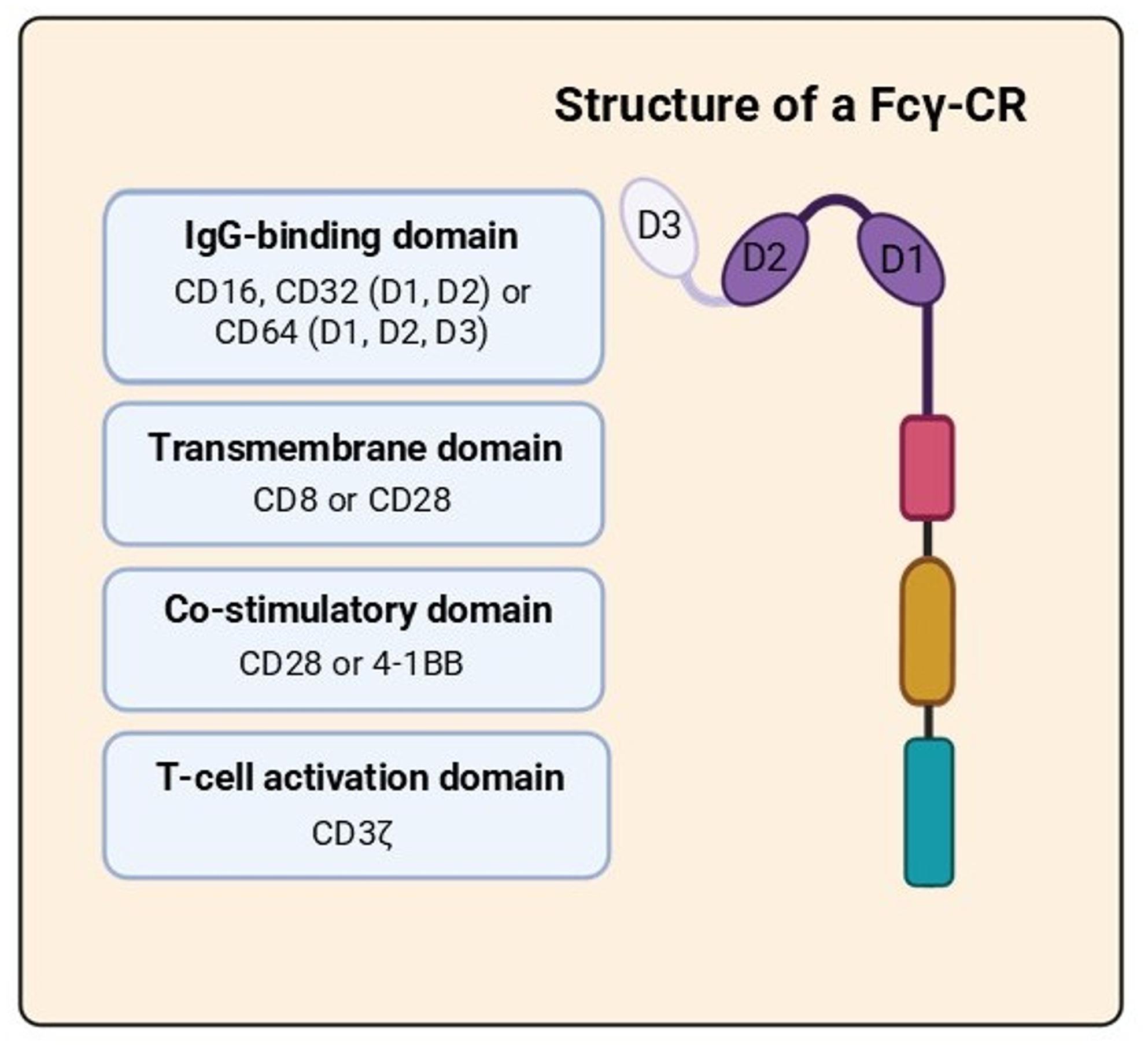
Modular structure of an Fcγ receptor–based chimeric receptor (FcγR-CR). This diagram illustrates the structure of an Fcγ chimeric receptor, which is expressed on T cells. The IgG binding site is located on one of the three Fcγ receptors (CD16, CD32, or CD64) and contains 2 or 3 immunoglobulin-like domains (D1 through D3) that recognize the Fc portion of antibodies bound to tumors. The extracellular IgG-binding domain is fused to the intracellular signaling modules via a transmembrane domain derived from either CD8 or CD28, which includes co-stimulatory domains such as CD28 or 4-1BB, along with the CD3ζ activation domain. This modular system allows activation of T cells in a manner dependent on antibodies for cytotoxicity, while providing the user with targeting options for any tumor-associated antigens targeted with FDA-approved monoclonal antibodies

FcγR-CR transducing T cells, modified with a synthetic construct that includes an extracellular Fcγ receptor (FcγR) domain, a transmembrane domain, and intracellular signaling domains such as CD3ζ, CD28, and 4-1BB (Fig. 3). This approach enables T cells to locate and respond to tumors covered with therapeutic mAbs. When these antibodies bind to the Fc region on tumor surfaces, they stimulate FcγR-CR T cells, which then produce cytokines and specifically destroy tumor cells [23, 47].

Different Fcγ receptors define the many forms of FcγR-CR T cells. CD16-CR T cells may detect IgG1 and IgG3 antibodies, including rituximab, trastuzumab, and cetuximab, expressing the FcγRIIIa (CD16) ectodomain. Due to their strong ADCC characteristics, extensive studies have been conducted on these cells [15]. On the other hand, CD32-CR T cells, which express the FcγRII (CD32) ectodomain, exhibit broader antibody specificity but may have lower ADCC activity compared to CD16-CR variants. Using the FcγRI (CD64) domain, which exhibits high affinity and low internalization, CD64-CR T cells can bind circulating IgG-opsonized antigens for extended periods [33, 72, 78, 82].

The CD16 ectodomain linked to a CD8α transmembrane domain is often used in the design for FcγR-CR T cells, followed by CD28 and CD3ζ as intracellular signaling modules (Fig. 3)**.** This design ensures efficient T cell activation by Fc-mediated contact with antibody-coated tumor targets, thereby fostering immunological synapse formation, cytokine release, and tumor killing [83].

Comprising a novel form of adoptive cell therapy, FcγR-CR T cells combine the specificity of mAbs with the potent cytotoxicity of T lymphocytes. Unlike conventional chimeric antigen receptor (CAR) T cells, which require identifying and engineering receptors suited to specific tumor antigens, FcγR-CR T cells function through a two-step process that enables greater adaptation to various antibody-opsonized targets. Targeting neoplastic cells using therapeutic mAbs defines the first stage of FcγR-CR T cell activity. Chosen for their sensitivity towards TAAs, these antibodies are proteins found on the surface of malignant cells. Typical examples of such TAAs are human epidermal growth factor receptor 2 (HER2), epidermal growth factor receptor (EGFR), and CD20, which are particularly targeted by trastuzumab, cetuximab, and rituximab, respectively [84]. These antibodies opsonize cancer cells and bind to specific antigens, thereby marking them for immunological recognition upon delivery. Since it provides the accuracy required for selective tumor destruction while sparing normal tissues, this targeting phase is crucial.

Therapeutic mAbs usually belong to the IgG1 or IgG3 subclasses, which are characterized by robust effector function and efficient interaction with FcγRs on immune cells. By using these antibodies on tumor cells, oncologists can direct FcγR-CR T cells to almost every tumor type for which a specific mAb is available or in development, thereby offering a modular, customized approach to cancer immunotherapy [33].

An essential first step in producing FcγR-CR T cells is to modify T cells to express synthetic receptors that can interact with and recognize the Fc portion of mAbs targeting specific TAA. The method starts with T cell extraction from the patient’s blood, then adds a synthetic receptor gene encoding the necessary FcγR-CR to these T cells. Lentiviral or retroviral vectors efficiently incorporate the genetic material into the T cell DNA when the gene is administered. Furthermore, tools used to add FcγR-CR genes at specified loci within the T cell genome for long-term expression are genome editing tools, such as CRISPR/Cas9. Following engineering, these FcγR-CR T cells are expanded and reinfused into the patient’s body, where they target tumor cells labeled with antibodies, thereby increasing the immune response through ADCC [85].

The next stage of the FcγR-CR T cell pathway starts when tumor cells are coated with mAbs. The Fc region of the bound antibodies is identified and attached to by the modified T cells expressing synthetic receptors with extracellular domains derived from FcγRs (e.g., CD16, CD32, or CD64). Functionally, this link resembles ADCC, a natural immunological process driven by NK cells and macrophages. T cells—known for their long-lasting persistence and high cytotoxicity—are included in the procedure in this intended system [86].

An immunological synapse forms between the T cell and the opsonized tumor cell following Fcγ binding. Usually, including CD3ζ for TCR-like activation and co-stimulatory domains like CD28 or 4-1BB (Fig. 3) to improve persistence and cytotoxic activity, this synapse enables signal transmission through the intracellular signaling domains encoded in the chimeric construct [87]. One significant benefit of FcγR-CR T cell therapy is its universality and flexibility. Unlike traditional CAR T cells, which require engineering to target a specific tumor antigen, FcγR-CR T cells can attack a wide range of malignancies by using different antibodies. This approach allows a single T cell product to be used with various antibody treatments, creating a plug-and-play, “off-the-shelf” platform [88]. This flexibility accelerates clinical development and simplifies manufacturing. It also enables quick adaptation to new targets as new mAbs are developed or approved. Additionally, FcγR-CR T cells can bind multiple tumor antigens simultaneously via distinct antibodies, potentially overcoming antigen diversity and resistance mechanisms common in solid tumors [89]. Moreover, T cell activity is controlled over time and space through antibody opsonization. Oncologists can influence T cell activation and killing by adjusting when and how much antibody is administered, thereby reducing off-target effects, including CRS, a common side effect of CAR T cell therapy [90].

Overall, FcγR-CR T cells offer a creative and flexible immunotherapy platform linking T cell-mediated cytotoxicity with mAbs treatment **(**Table 1**)**. Offering promise for various tumors, this approach may be a fundamental building block of next-generation immunotherapy since it uses a two-step process comprising antibody-mediated targeting and Fc receptor interaction.

**Table 1:** A comprehensive table summarizing different types of monoclonal antibodies (mAbs) used to treat various types of cancers, including their targets, mechanisms of action, and FDA-approved indications

Monoclonal antibody	Target antigen	Cancer type(s)	Mechanism of action	FDA-approved indication(s)
**Rituximab**	CD20	Non-Hodgkin lymphoma, CLL	ADCC, CDC, apoptosis induction	B-cell NHL, CLL
**Trastuzumab**	HER2/neu (ERBB2)	Breast, gastric, gastroesophageal	ADCC, inhibits HER2 signaling	HER2 + breast and gastric cancers
**Cetuximab**	EGFR	Colorectal, head & neck	Blocks EGFR signaling, ADCC	EGFR+ colorectal cancer, HNSCC
**Bevacizumab**	VEGF-A	Colorectal, NSCLC, glioblastoma, RCC	Inhibits angiogenesis	Colorectal, NSCLC, glioblastoma
**Daratumumab**	CD38	Multiple myeloma	ADCC, CDC, apoptosis induction	Multiple myeloma
**Atezolizumab**	PD-L1	NSCLC, urothelial cancer, TNBC	Immune checkpoint inhibitor	NSCLC, urothelial carcinoma, TNBC
**Nivolumab**	PD-1	Melanoma, NSCLC, RCC, HNSCC, HL	Immune checkpoint inhibitor	Various solid tumors and HL
**Pembrolizumab**	PD-1	Melanoma, NSCLC, CRC, etc.	Immune checkpoint inhibitor	Wide range of cancers, including MSI-high tumors
**Alemtuzumab**	CD52	CLL, T-cell lymphomas	ADCC, CDC, apoptosis	CLL (withdrawn from market but used under special programs)
**Brentuximab vedotin**	CD30	Hodgkin lymphoma, ALCL	ADC – delivers cytotoxic agent	HL, systemic ALCL
**Sacituzumab govitecan**	Trop-2	Triple-negative breast cancer (TNBC)	ADC – delivers SN-38 (topoisomerase inhibitor)	Metastatic TNBC
**Gemtuzumab ozogamicin**	CD33	Acute myeloid leukemia (AML)	ADC – delivers calicheamicin	CD33 + AML
**Ramucirumab**	VEGFR2	Gastric, NSCLC, colorectal, HCC	Anti-angiogenic	Gastric, NSCLC, colorectal, HCC
**Elotuzumab**	SLAMF7	Multiple myeloma	ADCC, NK cell activation	Relapsed/refractory multiple myeloma
**Ipilimumab**	CTLA-4	Melanoma, RCC, NSCLC	Immune checkpoint inhibitor	Melanoma, NSCLC, RCC

FcγR-CR T cells modified to express FcγRs have emerged from advances in genetic engineering and can target IgG-coated tumor cells. This approach leverages T cells’ strong cytotoxicity and antibodies’ natural specificity to provide a flexible framework for targeting multiple malignancies. Including FcγRs in T cells enhances their capacity to interact with tumor cells, increasing the potential for adoptive cell therapy [87].

The higher specificity and longevity of T cells relative to NK cells help explain why T cytotoxic cells are preferred over NK cells for generating FcγR-CR T cells. Although their function is less controlled and can be influenced by the immunosuppressive tumor microenvironment (TME), NK cells naturally generate FcγRs and support ADCC. On the other hand, T cells show greater adaptation, and their genetic changes help improve targeting precision. Moreover, T cells can survive longer within the TME, providing a continuous immune response necessary to destroy cancer cells. Given the lack of effective targeted treatments, researchers can enhance the cytotoxicity of the immune response against tumors by modifying T cells to express FcγRs, such as CD16a, CD32a, and CD64a [88].

FcγR T cells production consists of several steps. T cells are first obtained from the patient or a donor, then transduced with lentiviral vectors containing FcγR gene constructs. These vectors include the gene into the T cell genome, enabling stable FcγR expression on the T cell surface. Each of the central Fcγ receptors targeted—CD16a (FcγRIIIa), CD32a (FcγRIIa), and CD64a (FcγRI)—serves a different purpose in activating immune cells. While CD32a helps to remove immunological complexes, CD16a stimulates ADCC, and CD64a increases phagocytosis [44]. Following transduction, the T cells are grown ex vivo to ensure sufficient levels for injection. The altered T cells are then returned to the patient’s bloodstream and triggered by antibodies targeting tumor cells, boosting immune responses [89].

Endodomains were created by designing and building a CD16A-CAR with a hinge spacer and transmembrane region generated from CD8, along with the ζ-chain of the TCR/CD3 complex and the CD28 co-stimulatory signal transducer module (Fig. 3). This design ensures the activation and recruitment of PI3-kinase and signal transducers via the TCR ζ-chain contact, generating fully competent immune responses, including IL-2 production and target cell death [39, 90, 91].

Notwithstanding contradictory research on the relevance of these routes, growing evidence indicates that granule-independent cellular cytotoxicity is vital for in vivo tumor rejection [92, 93]. Two items of proof support the relevance of FasL-mediated cytotoxicity. Secondly, despite efficient TCR signaling, tumor-infiltrating CD8 + T cells often lack perforin-mediated cytotoxicity due to compromised release of cytolytic granules [94]. These results highlight the need for FasL-mediated cytotoxicity to eradicate vulnerable cancers after adoptive T-cell therapy. FcγR-CR-T cells that bind to the Fc fragment of mAbs on the surface of opsonized tumor cells may therefore show anti-tumor efficacy via granule-dependent and granule-independent cytotoxicity mechanisms, with these two systems fulfilling complementary, non-redundant roles for the efficient destruction of target cells [92, 93, 95].

Non-granular approaches include the release of chemokines and cytokines that draw additional immune cells to the tumor site, hence increasing the immune response. Working together, the two systems help to reduce cancer recurrence and improve tumor clearance [78, 96].

Various groups’ innovative studies have shown the viability of re-targeting T lymphocytes with Fcγ chimeric receptors (CD16A-CAR) to target opsonized tumor cells both in vitro and in vivo, therefore overcoming the restriction of using separate CARs for each possible tumor antigen [15, 97]. This advanced evolutionary design of CARs holds excellent promise, as it includes activities usually associated with therapeutic mAbs already in use in clinical settings, enabled by T-cell-dependent immune responses targeting the same antibody linked to cancer cells.

Previous studies showed that CD16A-CARs, equipped with different signaling domains, can redirect transduced T cells to respond to mAb-opsonized tumor cells, thereby triggering IL-2 release, proliferation, and target cell cytotoxicity—key features of a strong and self-sustaining immune response. In these studies, the CD16A (allotype V158) has been linked to the transmembrane and signaling domain of Fc epsilon receptor I gamma chain (FcεRIγ or FceRIγ) [97], the CD8α hinge and transmembrane domains, as well as the signaling domains of 4-1BB and CD3ζ[98], or the transmembrane and signaling domain of CD3ζ[15] **(**Fig.2). Structural changes in the CAR endodomains significantly affect signaling capacity and T-cell functions [99, 100]. 

Several previous studies support the idea that adoptive therapies meant to increase ADCC using T cells expressing CD16A-CAR may be very flexible, since they can improve the immune response via an antibody-dependent T-cell mechanism and maintain the biological and clinically relevant advantages of several therapeutic antibodies that can be given at any stage of the disease [101, 106].

Unlike antibody-derived CARs, which comprise an extracellular component consisting of a single-chain variable fragment (scFv) targeting a specific tumor-associated antigen, CD16A-CARs offer unique advantages [102]. Most scFv are derived from murine rather than human genes, which can trigger host immune responses, limiting their clinical use [102]. Moreover, most scFvs exhibit lower binding affinities for target antigens than monoclonal antibodies, which could compromise selectivity and efficient identification of tumor antigens. Therefore, arming T cells with FcγR-CR may significantly improve cancer treatment and appears appropriate to handle these potential issues. Therefore, it is essential to broaden the study to a human system of notable therapeutic relevance to explore the clinical possibility of this innovative technology.

In summary, FcγR-CR T Cells use a combination of cytotoxic mechanisms to target and destroy tumor cells tagged for destruction by the antibody. The cytotoxic processes used by FcγR-CR T cells are very similar to the processes used by NK Cells (natural killer Cells) for ADCC (Antibody Dependent Cytotoxicity) directed against antibody-coated tumor cells [59, 60]. When tumor-bound antibodies bind (through their Fc domain) to Fc Receptor (Fcγ receptors), they induce clustering of Fcγ receptors and activate the ITAM (Immunoreceptor Tyrosine Activation Motif)-dependent Signaling Pathways for cellular activation. Clustering and activation of the ITAM-dependent signaling pathway result in reorganization of the cytoskeleton and the production and release of cytotoxic effector molecules (CEMs) [67, 103]. Tumor cell death occurs predominantly via granule-dependent mechanisms involving perforin and granzymes. Granule-independent mechanisms, such as Fas-FasL interactions, may also kill tumor cells, but their role is more heavily dependent on cellular context and microenvironment [104, 105]. Several factors interact to affect cytotoxic pathways, including (but not limited to) the affinity of the Fcγ receptor, antibody isotype, antigen density on target cells, and local inflammatory signals. Collectively, these factors determine the total strength and efficacy of the overall cytotoxic response [67].

## Preclinical studies OF FcγR-CR T cells in solid tumor models

The development of FcγR-CR T cells marks a breakthrough approach in adoptive cell therapy, particularly for treating solid tumors. Unlike traditional CAR T cells, which are limited by fixed antigen specificity, FcγR-CR T cells use tumor-targeting mAbs to steer T cells toward a varied spectrum of TAAs. This flexibility offers a feasible choice for the varied and dynamic antigenic environment of solid tumors. Table 2 lists preclinical studies using FcγR-CR T cells in cancer models.

**Table 2:** Preclinical Studies Using FcγR-CR T Cells in Cancer Models

**FcγR construct**	**FcγR type**	**Antibody used**	**Target antigen**	**Cancer type**	**Model**	**Key findings**
CD16-CR T cells (first-gen)	CD16 (FcγRIIIa)	Rituximab	CD20	B-cell lymphoma	Raji xenograft in NSG mice	CD16-CR T cells showed redirected cytotoxicity only in the presence of rituximab; limited in vivo persistence due to lack of co-stimulation
CD16-CR T cells (second-gen with CD28 or 4-1BB)	CD16 (FcγRIIIa)	Trastuzumab	HER2	Breast cancer	SK-BR-3 xenograft in NSG mice	CD16-CR T cells + trastuzumab induced potent cytotoxicity against HER2+ cells; co-stimulatory domain improved persistence and function
CD32-CR T cells	CD32 (FcγRIIa)	Cetuximab	EGFR	Colorectal carcinoma	HT-29 xenograft	CD32-CR T cells + cetuximab induced strong ADCC and tumor inhibition in vivo
FcγRI-CR T cells	CD64 (FcγRI)	Daratumumab	CD38	Multiple myeloma	MM.1S xenograft model	FcγRI-CR T cells effectively killed CD38+ MM cells when coated with daratumumab; improved tumor control observed
CD16-CR T cells	CD16 (FcγRIIIa)	Atezolizumab (PD-L1)	PD-L1	NSCLC	A549 xenograft	CD16-CR T cells + atezolizumab reduced tumor growth, demonstrating checkpoint blockade synergy
CD16-CR T cells	CD16 (FcγRIIIa)	Cetuximab	EGFR	Head and neck SCC	FaDu xenograft	Enhanced tumor clearance with CD16-CR T cells + cetuximab, suggesting potential in solid tumors
CD32-CR T cells	CD32 (FcγRIIa)	Obinutuzumab	CD20	CLL	MEC1 xenograft	CD32-CR T cells worked synergistically with obinutuzumab, improving tumor cell lysis and survival

The studies covered here highlight that FcγR-CR T-cell platforms have a modular structure. While the studies indicate that no specific antibodies are higher order than their counterparts. Rather, the choice of antibody determines not only antigen specificity but also effector capacity, depending on which FcγRs are used to engage the Fc portion of each antibody. This modularity also creates a tunable system in which efficacy and safety are always related. This also means that FcγR-CR T cells will be affected differently compared with standard CAR-T cells and must therefore be developed through a systematic approach to clinical development.

The large variety of therapeutically approved monoclonal antibodies (mAbs) targeting different tumor-associated antigens in separate cancer types provides a strong foundation for developing Fc gamma receptor chimeric receptor (FcγR-CR) T cell therapies. Rituximab, Trastuzumab, and Cetuximab are examples of monoclonal antibodies (mAbs) that have shown efficacy through mechanisms including antibody-dependent cellular cytotoxicity (ADCC). FcγR-CR T cells can use their tumor-targeting specificity to redirect immune responses against cancerous cells. These cells can bind to the Fc region of monoclonal antibodies stuck to tumor cells by changing T cells to generate Fcγ receptors (like CD16 or CD32), hence enabling a flexible, modular approach for targeting several cancer kinds with a single modified T cell platform. This approach improves the flexibility of cell-based immunotherapies by simply changing the therapeutic antibody. It enables rapid adaptation to different tumor antigens, hence accelerating the transfer of FcγR-CR T cell therapies into clinical use.

### Breast cancer (HER2+, EGFR+, B7-H3+)

By opsonizing HER2-positive tumor cells, trastuzumab enables FcγR-CR T cells to have a notable cytotoxic impact. Repeatedly recorded in these models are tumor remission, lower metastatic load, and extended longevity (Table 2).

Because of its aggressive features and limited therapy options, triple-negative breast cancer (TNBC), defined by the absence of estrogen and progesterone receptors as well as HER2 expression, presents significant treatment challenges. TNBC may find a workable treatment approach in FcγR-CR T cells [25, 106–114].

Because FcγR-CR T cells can interact with tumor-targeting mAbs, which increases tumor cell detection and death, they are thought to be a potential therapy method for TNBC. Using modified expression of receptors, including CD16a (FcγRIIIa), CD32a (FcγRIIa), and CD64a (FcγRI), FcγR-CR T cells help to identify tumor cells covered in therapeutic antibodies, thereby generating strong immune responses. Combining FcγR-CR T cells with trastuzumab, cetuximab, and pembrolizumab—antibodies routinely used in cancer treatment—helps to increase their efficacy against TNBC cells. For patients with TNBC with few therapeutic options, cooperation between antibodies and modified T cells could improve therapy results [35, 40, 111].

Several TAAs have been identified in TNBC, such as B7-H3 and PD-L1, immune checkpoint proteins similarly raised in other cancers, including TNBC, and EGFR, a receptor usually overexpressed in TNBC cells [82, 117–121].

Directing FcγR-CR T cells toward tumor-associated antigens helps the immune system identify and destroy TNBC cells more effectively, lowering tumor burden and improving patient prognosis. Moreover, using Fcγ-CR T cells to target these antigens offers a possibility to solve the challenges related to the lack of specific, focused treatments for TNBC.

Overall, FcγR-targeted therapy offers great promise for improving TNBC treatment. By ADCC, immunological activation, and focused tumor cell elimination, engineering T cells to express FcγRs—including CD16a, CD32a, and CD64a—can increase the efficacy of antibody-based therapy. This approach could provide a fresh approach for TNBC, which now has few choices for effective treatment. More research is necessary to better understand the effects of receptor polymorphisms, enhance the development of FcγR-CR T cells, and refine therapeutic approaches for the pragmatic application of this potential treatment modality [82].

Recent preclinical studies have shown that FcγR-CR T cells have promise in TNBC models. Conversely, CD32a CR T cells improve T cell penetration and efficacy in the tumor microenvironment [107, 110, 115]. The findings show that CD32-CR T cells, HLA-independently, release CD107a and downregulate CD32 by selectively recognizing ligands on TNBC cells, including MDA-MB-468 and MDA-MB-231. While normal fibroblasts, myoblasts, and several epithelial and hematopoietic cancer cells are omitted, this cytotoxic activity includes HER2 + HCC-1954, CRC HT29, and glioblastoma U-373MG cells. The findings show that a particular type of BC cells mainly express CD32 ligands. The anti-tumor effectiveness of CD32-CR T cells is confirmed by several in vitro experiments, including confocal microscopy, bioluminescence, flow cytometry, and MTT. About three of seven BC cell lines responded to CD32-CR, suggesting the presence of distinct subpopulations of sensitive and resistant cells. As shown in earlier studies, where bispecific antibodies found CD44 as a receptor for lethal NK cells, biosensors like CD32-CR T cells are crucial in finding unknown surface chemicals [36, 64].

Notably, CD32-CR-mediated cytotoxicity appears independent of ADCC. The failure of human serum or IgGs to protect EGFR-positive BC cells, and the higher cytotoxicity observed in the presence of cetuximab, support this result. The findings support the idea that CD32-CR cytotoxicity targets ligands other than conventional antibody-binding sites. Especially at low effector-to-target ratios and with transduction rates below 100%, CD32-CR T cells show considerable anti-tumor effectiveness. Furthermore, CD32-CR T cells form distinct conjugates with sensitive BC cells, leading to a reciprocal cell destruction phenomenon absent in non-transduced (NT) T cells. This finding is similar to previous studies suggesting that NK cell exhaustion could be caused by CD16A downregulation in some epithelial and leukemic cells [37, 116, 117]. Although most hematological cancers are resistant, CD32-CR T cells mainly target breast cancer; specific colorectal and glioblastoma cells are susceptible. Based on their sensitivity, BC cell lines can be classified as sensitive or resistant. Transcriptomic analysis of these cohorts revealed 42 differentially expressed genes (DEGs), with ICAM1 standing out for its known roles in immune cell trafficking and its differential expression across breast cancer subtypes [36].

IgFc fragments and pentraxins are the ligands for FcγRII (CD32). It is still unknown whether other ligands are present. Connected to the intracellular CD28/ζ chain, CD32-CR T cells, including human chimeric receptors, were developed by the fusion of the extracellular region of CD32 and the transmembrane CD8α. Without the use of targeting antibodies, transduced T cells targeted three breast cancer and one colon cancer cell line among 15 total analyzed. Conjugation of BC cells with CD32-CR T cells produced CD32 polarization and down-regulation, CD107a release, mutual elimination, and proinflammatory cytokine production, which were not affected by human IgGs but were enhanced by cetuximab. CD32-CR T cells protected immunocompromised mice from the subcutaneous spread of MDA-MB-468 breast cancer cells. Analysis of RNA sequencing found a 42-gene pattern forecasting breast cancer cell sensitivity and favorable prognosis in advanced breast cancer.

Tyrosine-based activation motifs (ITAMs), while Intercellular Adhesion Molecule 1 (ICAM1) significantly controlled CD32-CR T cell-mediated cytotoxicity. CD32-CR T cells could help find cell surface CD32 ligands and new prognostically relevant transcriptome signatures, and develop ground-breaking breast cancer therapies [36]. Fcγ-CRs activation sends signals via immunoreceptor CD32 and CD32C via ITAMs in their cytoplasmic tails [71]. CD64 and CD16A are dimers that comprise a ligand-binding domain and a signal-transducing γ-chain bearing an ITAM. Since it is found on natural killer (NK) cells and specific subsets of monocytes and NK T cells, CD16A is especially crucial for ADCC [118–120]. Although NK cells are effective in causing ADCC, their presence in solid tumors remains limited, even with the overexpression of activating ligands such as MICA/B, and their infiltration shows no significant prognostic relevance [37, 116, 121–123]. Conversely, CD8 + T cells are more common in cancers and are associated with better clinical outcomes.

In preclinical work already affecting clinical projects, Cui et al. (2023) reported on T cells engineered to express a high-affinity version of CD64 known as CFR64. This preclinical result presents a strong argument for the clinical deployment of CD64-CAR T cells, particularly when sustained antibody binding and prolonged T-cell engagement are critical [124].

### Colorectal cancer (EGFR+)

Characterized by the overexpression of EGFR in a significant number of patients, colorectal cancer (CRC) remains a serious worldwide health concern. EGFR-targeted treatments, including mAbs such as cetuximab and panitumumab, have suppressed tumor growth. Still, alterations in downstream signaling pathways, such as KRAS, often compromise their efficacy, leading to resistance. Using altered T cells that express FcγRs has been explored to address this. Examined for their ability to increase anti-tumor reactions in EGFR-positive colorectal cancer are polymorphic variants such as CD16^158F^, CD16^158V^, CD32^131H^, and CD32^131R^. Notable are FcγRIIIa (CD16) and FcγRIIa (CD32), whose polymorphisms influence IgG antibody affinity. The CD16^158V^ variant shows more IgG1 and IgG3 affinity than the CD16^158F^ variant. The CD32^131H^ variant shows more IgG1 affinity than the CD32^131R^ variant. The differences can significantly affect the efficacy of therapeutic antibodies relying on ADCC pathways. Using information on FcγR polymorphisms, scientists have altered T cells to express CRs that include these variations. Arriga et al. (2020) investigated T cells transduced with CD16^158F^-CR, CD16^158V^-CR, CD32^131H^-CR, and CD32^131R^-CR. When interacting with KRAS-mutated HCT116 colorectal cancer cells, T cells expressing CD16^158V^-CR and cetuximab showed significantly higher levels of IFNγ and TNFα. Moreover, these altered T cells in vivo controlled tumor growth, suggesting a possible way to overcome resistance in KRAS-mutated, EGFR-positive colorectal cancer [22].

FcγR polymorphisms’ therapeutic relevance includes altered T cells and the natural immune response in patients undergoing anti-EGFR mAbs treatment. Studies have indicated that people with the FcγRIIIa-158 V/V genotype show a more favorable response to cetuximab, characterized by improved progression-free survival (PFS). This suggests that the natural affinity of FcγR mutations for therapeutic antibodies could influence treatment results [125].

Including FcγR polymorphism changes into altered T cells is a creative way to increase the efficacy of EGFR-targeted therapies in colorectal cancer. Especially in KRAS-mutated cancers, using the improved affinity interactions of some FcγR variants, such as CD16^158V^, with therapeutic antibodies can increase ADCC and overcome resistance mechanisms [126].

Examining a novel approach for treating KRAS-mutated colorectal cancer (CRC) cells, Arriga et al. (2020) find these cells are usually resistant to epidermal growth factor receptor (EGFR)-targeted monoclonal antibodies like cetuximab. To evaluate their effectiveness in overcoming this resistance, the researchers changed T cells to express multiple polymorphic FcγR-CRs, namely CD16^158F^, CD16^158V^, CD32^131H^, and CD32^131R^. Relative to those with CD16^158F^-CR and CD16^158V^-CR, T cells transduced with CD32^131H^-CR and CD32^131R^-CR showed increased expression levels. Only CD32^131R^-CR T cells showed effective binding to panitumumab and soluble cetuximab among the designs. Still, the CD16^158V^-CR T cells and cetuximab produced significant amounts of tumor necrosis factor-alpha (TNFα) and interferon-gamma (IFNγ), suggesting strong immunological activation. This combination also efficiently eliminated KRAS-mutated HCT116 and A549 cancer cells in vitro. Using a CB17-SCID animal model, the combination of cetuximab and CD16^158V^-CR T cells subcutaneously controlled the growth of HCT116 tumors. The results show that EGFR-specific mAbs can steer T cells altered with the CD16^158V^ chimeric receptor to target and destroy EGFR+ KRAS-mutated colorectal cancer cells, offering a possible path for the creation of new immunotherapies for resistant colorectal cancers [127].

### Pancreatic cancer (EGFR+, Mesothelin)

Pancreatic ductal adenocarcinoma, known for its poor prognosis and immunosuppressive microenvironment, has also been a target for FcγR-CR T cells. CD64-CR T cells combined with anti-EGFR antibodies (e.g., cetuximab) or anti-mesothelin mAbs have enhanced tumor cell killing in 3D spheroid cultures and orthotopic models. CD64 offers higher affinity Fc binding, improving the sensitivity of opsonized targets and recycling capacity [128].

Mainly when characterized by the overexpression of antigens like EGFR and mesothelin, CRCs and pancreatic cancers provide significant treatment challenges. mAbs aimed at these antigens have been developed to increase anti-tumor reactions. Their interaction with Fc gamma receptors (FcγRs) on immune cells, which support ADCC, determines the efficacy of these mAbs. CD16^158F/V^ and CD32^131H/R^, polymorphisms in FcγRs, could significantly affect this interaction and treatment outcomes [129].

Part of cetuximab’s effectiveness depends on ADCC driven by FcγRs. Studies show that patients with the CD16 158 V/V genotype respond better to cetuximab, likely because of increased NK cell-mediated ADCC. By contrast, those with the CD16 158 F/F genotype can show lower reactions, stressing the therapeutic relevance of FcγR polymorphisms in CRC treatment [130, 131].

Often overexpressing EGFR and mesothelin, pancreatic ductal adenocarcinoma (PDAC) makes both antigens excellent targets for mAb therapy. The dense stromal environment and immunosuppressive properties of PDAC hamper effective therapy. Unlike in CRC, the relevance of FcγR polymorphisms in PDAC is less well defined. People with high-affinity FcγR variants, such as CD16^158V^, may have improved reactions to mAb treatment directed against EGFR or mesothelin because of more efficient ADCC. More research still needs to clarify these links and their potential therapeutic effects. Immunotherapy advancements have led to the development of CAR T cells altered to show FcγRs, enabling them to find and destroy cancer cells covered with therapeutic antibodies. Compared to those expressing the 158 F variant, T cells expressing the CD16^158V^ variant show better cytotoxic activity against tumor cells in the presence of mAbs. This approach can overcome tumors having particular antigenic features, such as EGFR+ colorectal cancer and mesothelin-expressing pancreatic ductal adenocarcinoma [132].

### Prostate cancer (PSMA)

Often characterized by the overexpression of Prostate-Specific Membrane Antigen (PSMA), prostate cancer is among the most common malignancies in men. Predominantly found in prostate cancer cells, PSMA is a type II membrane glycoprotein that has been increasingly important for cancer treatment. Recent advances in immunotherapy, particularly in CAR T cell therapy, have shown promise for targeting PSMA in prostate cancer [133]. FcγR-CR T cells can engage with antibody-coated tumor cells utilizing Fcγ receptor binding when coupled with mAbs targeting particular TAAs, such as PSMA in prostate cancer [17, 98]. Unlike traditional CAR T cells that need direct tumor antigen recognition by the T cell receptor, FcγR-CR T cells target tumors using the specificity of mAbs, hence offering a more remarkable ability to address antigenic heterogeneity in cancer cells.

Zhang et al. (2020) investigated the efficacy of FcγR-CR T cells in combination with anti-PSMA antibodies using immunodeficient mouse models. The results showed that without causing off-target damage, Fcγ-CR T cells efficiently targeted PSMA-expressing cancer cells, significantly lowering tumor load. The tumor regression was especially linked to a rise in T cell infiltration at the tumor location, suggesting that the anti-PSMA antibodies effectively guided FcγR-CR T cells to the tumor microenvironment. The results fit the known reality that FcγR-CR T cells can provoke strong anti-tumor reactions by a mechanism similar to that of NK cells in ADCC [133].

A significant finding in these preclinical studies is the lack of off-target damage in immunodeficient mice. One major difficulty with immune therapies like CAR T cells is the potential for collateral damage to healthy tissues caused by cross-reactivity with normal cells. The particular interaction between the Fc region of the antibody and the Fcγ receptors on the altered T cells, FcγR-CR T cells, coupled with anti-PSMA monoclonal antibodies, show selective targeting. This focused approach increases its possible clinical use by reducing the danger of harming non-cancerous cells [133].

Another important element is the immunological memory generated by FcγR-CR T cells. Tumors rechallenge studies in preclinical environments suggested that FcγR-CR T cells could produce lasting immunological responses. This indicates that T cells proficiently target and eradicate the tumor during the initial therapy and may protect against subsequent tumor recurrences [133]. The preclinical efficacy of FcγR-CR T cells in prostate cancer models, mainly when used with anti-PSMA mAbs, has significant potential for therapeutic applications. Ongoing clinical trials are currently investigating CAR T cell therapeutics aimed at PSMA [134], whereas incorporating FcγR-CR T cells may offer an alternate or supplementary strategy. The capacity of FcγR-CR T cells to utilize current mAbs, such as those directed against PSMA, may expedite the advancement of tailored therapies for prostate cancer patients.

Subsequent clinical investigations must further assess the safety, effectiveness, and optimal dosing protocols for FcγR-CR T cells in combination with monoclonal antibodies, such as anti-PSMA. Furthermore, research exploring the integration of FcγR-CR T cells with immune checkpoint inhibitors (such as anti-PD-1 or anti-CTLA-4) may enhance therapeutic efficacy by mitigating the immunosuppressive tumor microenvironment [133].

The adaptability of the FcγR-CR T cell strategy, along with the application of established mAbs, presents a potentially revolutionary therapy for individuals with prostate cancer. Although additional research is necessary, especially in humanized models and clinical trials, the encouraging results from animal studies suggest that FcγR-CR T cells may be crucial for the future of prostate cancer immunotherapy. While PSMA-targeted CAR T cells are now being clinically studied, animal models have shown promise for FcγR-CR T cells paired with anti-PSMA monoclonal antibodies. In immunodeficient mice, these studies demonstrate efficient targeting of PSCA-expressing tumor cells, reduced tumor burden, and no off-target damage [133].

### Lung cancer (EGFR+, PD-L1+)

Non-small cell lung cancers (NSCLC) that express EGFR have been targeted using CD16-CR T cells and cetuximab, resulting in significant anti-tumor effects both in vitro and in vivo. Studies have assessed CAR T and FcγR-CR T cells with anti-PD-L1 antibodies to simultaneously target tumor cells and alter the immunosuppressive milieu [135, 136].

In lung cancer, FcγR-CR T cells could be paired with monoclonal antibodies targeting proteins often overexpressed in NSCLC, including epidermal growth factor receptor (EGFR) and mesothelin. Several clinical studies have shown that cetuximab (anti-EGFR) and mesothelin-targeting antibodies are effective [137]. Still, resistance mechanisms often arise, underscoring the need for novel strategies such as Fcγ-CR T cells to enhance therapeutic outcomes.

The immunological milieu in NSCLC is often immunosuppressive and characterized by myeloid-derived suppressor cells, regulatory T cells, and other immune-evasive mechanisms. These challenges reduce the effectiveness of traditional immunotherapies, such as ICIs [138]. On the other hand, FcγR-CR T cells can overcome these challenges by directly interacting with antibody-coated cancer cells. By modifying T cells to express high-affinity Fcγ receptors in a tumor-specific manner, researchers hope to improve their longevity, cytotoxicity, and overall efficacy.

Studies have shown that CD16-chimeric T cells, when combined with cetuximab, can effectively target EGFR-positive tumor cells in vitro and in preclinical animal models [21]. The changed T cells showed better cytotoxicity and a longer immune response than unmodified T cells. The emergence of resistance to standard treatments, such as ICIs and mAbs, poses a significant challenge to the management of NSCLC. Due to resistance to ICIs, particularly those targeting PD-1/PD-L1, managing NSCL has become increasingly complex [137]. In NSCLC, the overexpression of immunological checkpoint molecules, such as PD-L1, on tumor cells helps shield them from T cell-mediated destruction. In this context, FcγR-CR T cells can avoid the need for direct recognition by T cell receptors by targeting tumor cells through their interaction with the Fc region of monoclonal antibodies.

Though promising preclinical results have sparked interest in this strategy, the clinical use of FcγR-CR T cells in lung cancer therapy is still in its early stages. Current clinical trials are evaluating the safety and efficacy of FcγR-CR T cells in combination with several mAbs in NSCLC and other cancers [139]. Furthermore, tailored therapeutic strategies may enhance the effectiveness of FcγR-CR T cells. Genetic variations in FcγR genes, particularly CD16, might influence the effectiveness of antibody treatment and the outcomes of FcγR-CR T cell treatments [21].

### Head and neck squamous cell carcinoma (HNSCC, EGFR+)

From the mucosal epithelium of the oral cavity, throat, and larynx, head and neck squamous cell carcinoma (HNSCC) is a varied group of cancers. Despite advances in surgical, radiotherapeutic, and chemotherapeutic treatments, the five-year survival rate for advanced head and neck squamous cell carcinoma (HNSCC) stays low, primarily due to delayed diagnosis, treatment resistance, and immune evasion by neoplastic cells. Roughly 90% of HNSCC cases overexpress the epidermal growth factor receptor (EGFR), which is associated with a worse prognosis and is therefore a primary therapeutic target [140].

Although their clinical effectiveness is limited in many patients due to insufficient immune activation, mAbs such as cetuximab have been approved for the treatment of HNSCC. Immunotherapeutic strategies that boost ADCC responses are being studied to improve outcomes, particularly using FcγR-CR T cells, especially those expressing CD16 (FcγRIIIa) [138]. Though T cells lack natural FcγRs, NK cells naturally mediate ADCC via CD16. Using CD16 or other FcγR ectodomains combined with intracellular signaling domains such as CD3ζ and co-stimulatory components such as CD28 or 4-1BB, researchers have created chimeric receptors that endow T cells with ADCC-like properties [132, 141].

By improving the ADCC effectiveness of cetuximab, CD16-CR T cells offer a solution to these limitations. When used with cetuximab, CD16-CR T cells have been shown in preclinical studies to elicit strong antitumor responses against EGFR+ cancers. T lymphocytes altered with CD16 chimeric receptors were effectively stimulated by cetuximab-coated EGFR+ cancer cells, showing notable cytotoxicity and cytokine generation, according to Kudo et al. (2014) [142]. Polymorphisms changing the FcγR’s ability to bind IgG alter its functional affinity. The CD16^158V^ variant shows greater affinity for IgG1 than CD16158F, thereby improving its ADCC activity. Caratelli et al. (2020) studied T cells expressing CD32A^131R^ or CD16^158F^-CRs in vitro versus EGFR+ tumor cells under cetuximab or panitumumab. Although the low-affinity variants can still cause cytotoxicity, the high-affinity CD16^158V^ variant showed better tumor lysis and cytokine generation, supporting its use in Fcγ-CR systems [21].

FcγR polymorphisms have been linked to clinical responses to cetuximab in HNSCC patients. People with the CD16 158 V/V genotype are more likely to show clinical improvement, suggesting that genetically altered T cells expressing this variation could improve treatment effectiveness in broader patient populations [140].

FcγR-CR T cells might help reshape the tumor microenvironment and target opsonized neoplastic cells. Activated T cells generate pro-inflammatory cytokines such IFN-γ and TNF-α, which help antigen presentation and draw additional immune cells. FcγR-CR T cells could increase T cell infiltration and reduce immune suppression in HNSCC when used with other immunotherapies, including immune checkpoint inhibitors (ICIs) [137].

Moreover, Fc-engineered antibodies showing increased FcγR binding affinity (e.g., afucosylated cetuximab variants) can increase the effectiveness of CD16-CR T cells even more [143]. The synergies suggest that combinatorial immunotherapy in HNSCC may be fundamentally based on FcγR-CR T cells.

### Glioblastoma (EGFRvIII, IL13Rα2)

The survival of glioblastoma cancer stem cells (GSCs), which show inherent resistance to cytotoxic treatments and can self-renew and repopulate tumors, is a key factor in GBM recurrence and treatment failure [1, 6].

A key factor influencing the biological behavior of GSCs is spatial heterogeneity. While those from the invasive peritumoral areas (p-GSCs) display less proliferation and a more dormant state, those from the central tumor core (c-GSCs) exhibit increased proliferative and tumorigenic potential [153, 154]. These inherent differences have significant therapeutic implications, as c-GSCs tend to promote tumor growth and recurrence, whereas p-GSCs are thought to facilitate invasion into neighboring normal brain tissue [155].

The epidermal growth factor receptor (EGFR), which is often overexpressed or mutated (e.g., EGFRvIII) in GBM, has emerged as a promising treatment target. EGFR dysregulation causes oncogenic signaling, treatment resistance, and poor prognosis in GBM patients [144–147].

Activating immunological effector cells, such as CD16 (FcγRIIIa), which interacts with the Fc region of IgG1 antibodies, enables ADCC [157, 158]. There are two naturally occurring polymorphic forms of CD16: CD16-158 V (high-affinity) and CD16-158 F (low-affinity) [136, 157]. These variants differ in their ability to bind the Fc region of IgG1 antibodies, including cetuximab, an FDA-approved anti-EGFR mAb.

Genetically engineered T cells expressing CD16^158V^-CR or CD16^158F^-CR were evaluated for cytotoxic activity in the presence of cetuximab (IgG1) or panitumumab (IgG2). CD16^158V^-CR T cells and cetuximab demonstrated notable cytotoxicity against EGFR + c-GSCs, as evidenced by caspase-3 activation and the release of pro-inflammatory cytokines, including TNF-α and IFN-γ [160]. On the other hand, CD16^158F^-CR T cells or CR T cells treated with panitumumab would not be able to promote efficient ADCC, as IgG2 antibodies do not bind well to CD16, and the 158 F mutation has a lower affinity [21, 84, 148, 149].

The p-GSCs were resistant to CD16-CR/cetuximab-induced cytotoxicity, with lower EGFR expression. This emphasizes the need for antigen density and spatially distinct cancer stem cell traits to affect therapy results. Moreover, the focused strategy on c-GSCs—responsible for the tumorigenic core—could offer significant clinical benefits, particularly in reducing recurrence triggered by these aggressive subpopulations [144, 150, 151].

To address the challenges posed by the blood-brain barrier (BBB), this work builds on an expanding body of research demonstrating the feasibility and safety of CAR- and CR-T cell therapies for brain tumors, including intracerebroventricular and intratumoral delivery. Chemokine receptors and adhesion molecules—including integrins (e.g., VLA-4) and the CXCL10–CXCR3 axis—may help modified T cells migrate effectively to intracranial tumors by increasing T cell homing to central nervous system sites of inflammation. Monoclonal Abs used in conjunction with Fc-engineered CAR T cells offer several advantages: (i) flexibility in target antigen choice using antibody interchange; (ii) precisely controlled effector function using affinity-modified Fc receptors; and (iii) the potential for combination immunotherapies that enhance efficacy while lowering systemic toxicity. Cetuximab’s (IgG1) dependency emphasizes the need for Fc-FcγR interactions in enabling efficient ADCC [21, 148, 152].

This work shows that CD16^158V^-CR T cells and cetuximab might particularly and efficiently destroy EGFR-expressing c-GSCs. Especially in EGFR+ tumors, this approach might offer a therapeutically relevant option for enhancing GBM therapy outcomes by leveraging ADCC. Apart from looking at combination treatments with checkpoint inhibition or radiation [153–155]. Future studies are required to assess in vivo effectiveness, blood-brain barrier permeability, and immune-mediated toxicity.

Preclinical studies consistently show Fcγ-CR T cell-mediated tumor control in immunodeficient mouse models using high-dose antibody therapy (which is typically not used at the dose or concentration levels in patients). Thus, preclinical results from these types of model systems cannot be directly translated into human solid tumors. Additionally, while antigen density, antibody isotype selection, and FcγR affinity can be optimized in preclinical models, these characteristics may differ significantly from those observed in a clinical setting. These differences demonstrate a clear need for more physiologically relevant preclinical models that integrate endogenous FcγR-expressing immune cells and evaluate competing fc engagement.

## Clinical translation of engineered FCγ-CR T cells

Engineered Fc gamma receptor (FcγR)-CR T cells create a flexible and possibly universal immunotherapeutic platform using the broad availability of tumor-targeting mAbs. Through an antibody-dependent mechanism, chimeric versions of FcγRs—CD16a (FcγRIIIA), CD32a (FcγRIIA), or CD64a (FcγRI)—enable the redirection of T cells to various tumor antigens. This approach overcomes the fixed specificity limitation of conventional CARs and enables real-time adaptability in targeting TAAs.

Many clinical studies **(**Table 3**)** on the safety and effectiveness of FcγR-based CAR T cells have started. Patients with adult T-cell leukemia (ATL) participated in a groundbreaking clinical trial evaluating CD16a-based CAR T cells. This work combined Mogamulizumab, an anti-CCR4 monoclonal antibody, with CD16a (158 V)-CD3ζ chimeric receptor T cells [156]. The trial showed early signs of anticancer activity and suggested that the altered T cells were well tolerated and could enhance the antibody’s therapeutic efficacy.

**Table 3:** Clinical Trials Involving FcγR-Related Strategies in Cancer Therapy

Trial identifier	Phase	Study title	Therapeutic strategy	Cancer type(s)	Status	Key findings
NCT02776813	Phase 1	CD16-CR T Cells Combined with Rituximab in B-cell Malignancies	CD16-CR T cells + Rituximab	B-cell lymphomas	Completed	Safety established; preliminary efficacy observed
NCT03266692	Phase 1	CD16-CR T Cells with Trastuzumab in HER2 + Solid Tumors	CD16-CR T cells + Trastuzumab	HER2 + breast and gastric cancers	Recruiting	Ongoing; results pending
NCT03189836	Phase 1	CD16-CR T Cells and Cetuximab in EGFR+ Cancers	CD16-CR T cells + Cetuximab	Colorectal and head & neck cancers	Active, not recruiting	Awaiting data analysis
NCT03874897	Phase 1	Chimeric Antigen Receptor T Cells Targeting Claudin18.2 in Solid Tumors	CAR-T cells targeting Claudin18.2	Gastric and pancreatic cancers	Recruiting	Early-phase study; safety and efficacy to be determined
NCT04219254	Phase 1/2a	BI-1206 (Anti-CD32b) with Pembrolizumab in Advanced Solid Tumors	Anti-CD32b antibody + Pembrolizumab	Various advanced solid tumors	Active, not recruiting	Preliminary data suggest potential to overcome anti-PD-1 resistance

Several solid tumors have been evaluated with CD16a-CAR T cells in Phase I research (NCT03266692). This trial used CD16-CR T cells with SEA-BCMA in Relapsed or Refractory Multiple Myeloma.

A clinical trial (NCT03189836) used a novel approach in B-cell malignancies by administering CD16-CAR T cells alongside Rituximab, a mAb targeting CD19. This study has shown growing clinical interest in using antibody-CAR synergy for hematologic malignancies (ClinicalTrials.gov, NCT03189836).

A clinical trial (NCT04219254) has shown anticancer activity of CD32a-based CAR T cells combined with Pembrolizumab in NSCLC and various epithelial tumors.

Studies on high-affinity CD16 polymorphisms have sought to increase CAR activity. Rataj et al. (2019), showed that high-affinity CD16^V158^ CARs, when combined with Fc-engineered antibodies, greatly enhanced in vitro proliferation and cytotoxicity, affecting present efforts to use these constructs in solid tumor settings [132].

The development of FcγR-CR T-cell platforms has been led by insights from NK cell-based treatments and their interactions with FcγRs. Hamilton and Plangger (2021, Biologics) and Gemelli et al. (2022) have highlighted the need of FcγR interaction in improving the effectiveness of ADCC in lung cancer, which parallels the basic mechanism used by FcγR CAR T cells in clinical trials [139].

Modified FcγR-CR T cells are being clinically translated more quickly. Clinical trials using CD16a, CD32a, and CD64a as extracellular domains in CAR T cells are now exploring treatments for hematologic malignancies and solid tumors, including ATL, breast cancer, NSCLC, and HNSCC. These studies often use antibody-based targeting strategies, offering a flexible and modular approach to adoptive T-cell therapy. Though most of these projects are still in early testing, the growing clinical data underscore the viability, safety, and potential effectiveness of FcγR-based CAR systems across several cancer types.

We focused the study-by-study descriptions on the preclinical and translational analysis into short summaries allowing for the inclusion of the integrative synthesis at the end of the preclinical analysis so that each of the experimental limitations encountered in each of the studies can be evaluated collectively; as follows: reliance upon immunodeficient model systems, supraphysiologic doses of monoclonal antibodies, limited assessment of Fc competition on tumor cells, and the variability (both temporal and quantity) of antigen density or binding affinity for FcγR. The clinical and safety evaluations have been expanded to provide an up-to-date, comparative assessment of cytokine release syndrome, immunogenicity, and Fc-mediated competition in FcγR-CR T-cell therapy compared to approved immunotherapies using cell-based products. Autologous CAR-T cell products provide extremely tumor-specific targeting but also have the highest incidence and severity of CRS and are at greatest risk due to the potent activation and time demands associated with their manufacture. Allogeneic NK-cell therapies have favorable safety profiles but limited persistence and efficacy duration following infusion. In contrast, FcγR-CR T-cells occupy an intermediate position in the translational hierarchy, as they provide both antibody-mediated and dose-dependent modulation of effector activity. However, the binding of Fc receptors to target antigen may also enhance cytokine release and result in Fc competition with endogenous FcγR-expressing immune cells. Furthermore, the modular approach to targeting FcγR-CR T cells via sequential or combinatorial administration of monoclonal antibodies raises concerns about antibody format, receptor design, and systemic exposure, given the potential for unintended targeting of off-tumor tissues.

## Advantages of FCγR-CR T cell therapy

CD16-CR T cells combined with TAA-specific mAbs have provided clear evidence of antitumor activity toward hematologic and epithelial malignant cells in vitro and in vivo. The mechanisms by which CD16-CR T cells, in combination with mAbs, eliminate cancer cells involve both granule-dependent and granule-independent cellular cytotoxicity. This information represents a platform for developing FcγR-CR-based targeted therapies of virtually any malignancy, as long as therapeutic mAbs with the appropriate specificity are available. To reach this goal, additional information is needed to optimize FcγR-CR and mAb combination before testing the described strategy in a clinical setting. To this end, investigators will need to accomplish three primary tasks. The first task will be to identify strategies to expand the pool of therapeutic mAbs capable of redirecting T cells against cancer cells. The second task should aim to determine the solid tumor to be targeted. The third task should determine the in vivo toxicity of FcR-CR-based immunotherapy. In this context, it is critical to consider the basis for the interaction of therapeutic mAbs with Fcγ receptors. The IgG1 isotype is the most prevalent IgG mAb subclass used in the clinic **(**Table 1). IgG1 mAbs preferentially bind CD16^v158V/V^ but also CD16^158V/F^ variants. They can trigger NK cell- and monocyte-mediated ADCC (antibody-dependent cellular cytotoxicity) and complement-dependent cytotoxicity. In contrast, IgG2 is also used in mAb therapeutics; however, it elicits significantly weaker ADCC because it does not trigger NK cell-mediated cytotoxicity compared to IgG1. Nevertheless, they can still elicit a myeloid cell-mediated cytotoxicity [41]. As a consequence, the use of IgG2 therapeutic mAbs in EGFR-positive malignancies, and particularly in those with KRAS mutations, may trigger lower antitumor activity than IgG1 mAbs. Interestingly, most subclasses of monomeric mAbs, including IgG1, IgG3, and IgG4 but not IgG2, bind FcγRI (CD64) with high affinity, leading to potent myeloid-mediated cytotoxicity and phagocytosis. Unfortunately, CD64-mediated ADCC may have only a limited impact on inhibiting tumor progression, as it does not involve NK cells. Given the availability of four IgG2 and three IgG4 therapeutic mAbs, it is likely that investigators will maximize the antitumor effect of the therapeutic mAb of interest by engineering T cells with the most appropriate FcγR-CR. Since the CD16-CR is the only molecule available in laboratories today for identifying the best combination of therapeutic mAb with FcγR-CR, it will be necessary to develop the CD32 and CD64 CRs for use in additional in vitro and in vivo studies.

It is noteworthy that solid tumors are not an ideal target for immune cells. This is mainly due to cancer cells’ ability to evade immune cells through multiple mechanisms that affect the accessibility, persistence, and function of immune cells in the tumor microenvironment. Compelling evidence suggests that solid tumors do not promote efficient ADCC, as their microenvironment is deficient in NK cells [42] but rich in M2 macrophages endowed with immunosuppressive and pro-angiogenic functions [43] and regulatory T cells [44]. Therefore, such a hostile microenvironment favors tumor progression. Nevertheless, colorectal carcinoma (CRC) represents an interesting exception. Among solid tumors, immune cell infiltration in the CRC microenvironment is associated with improved overall survival even in the presence of known immunosuppressive cells such as tumor-associated macrophages [45, 46], and regulatory T cells [44]. In addition, two lines of evidence suggest that ADCC contributes to the antitumor activity of anti-EGFR mAbs. First, a subset of CRC cells is consistently infiltrated by NK cells [132]. Second, a CD16^158V/V^ genotype predicts favorable clinical outcomes in CRC patients [32]. Based on this information, CRC could be an ideal target for assessing the antitumor activity of FcγR-CR T cells.

Finally, to determine the potential toxicity of this treatment, it may be useful to use immunocompetent mice bearing a spontaneous or engrafted CRC, targeted with mouse FcγR-CR T cells in combination with mouse anti-EGFR mAbs.

Among the various FcγR-CR designs, CD64-based CRs show greater affinity for IgG subclasses, enabling better antibody binding and recycling. Even with low antibody concentrations, this improves antigen identification. In models of HER2+, EGFR+, and CD20 + cancers, CD16-CR T cells have been more thoroughly tested and validated. With many models showing more than a 70% decrease in tumor size, improved overall survival, and the induction of immunological memory in rechallenge testing, preclinical investigations frequently achieve notable success rates [133]. The immunosuppressive tumor microenvironment, physical infiltration barriers, and antigen heterogeneity may restrict efficacy. Moreover, therapeutic translation must be carefully evaluated for potential off-target effects and systemic cytokine production.

Growing preclinical data suggest that FcγR-CR T cells, particularly those that use the CD16 or CD64 domains, offer a strong and flexible treatment option for solid tumors. Their compatibility with approved mAbs offers an interesting clinical route for repurposing current drugs alongside creative cell therapies.

Initial clinical trials are currently underway to assess FcγR-CR T cells in combination with rituximab (CD20 + lymphomas), cetuximab (EGFR+ malignancies), and trastuzumab (HER2 + tumors) (Moyes et al., 2022). These studies will assess the durability of response, persistence, dose regimens, tolerability, and safety.

The modular design, which enables rapid target switching, and the positive preclinical efficacy across many tumor types, justify advancing to clinical trials. However, further studies using immunocompetent animals and humanized murine models are vital to thoroughly evaluate potential adverse effects and improve safety characteristics before broad clinical use.

Engineered FcγR-CRCR T cells offer a universal targeting system that allows targeting any tumor by just changing the supplied monoclonal antibody. This modular approach provides a flexible, time-efficient therapy option, eliminating the need to reengineer CAR T cells for each tumor-associated antigen.

One significant advantage of this approach is the potential to reduce antigen escape. Therapeutic resistance is often caused by loss or downregulation of a single target antigen in conventional CAR T therapy. FcγR-CR T cells let antibodies be quickly replaced to target extra or alternative antigens, solving tumor heterogeneity and antigen loss.

These altered T cells show notable combinatorial synergy with various immunotherapies. They might, for example, be co-administered with oncolytic viruses, immune checkpoint inhibitors, or bispecific antibodies to increase general anticancer activity. This helps create integrated multimodal cancer drugs that mutually improve immune reactions.

Notably, FcγR-CR T cells offer better control and safety. Doctors can control T cells’ activity in vivo by adjusting the dosage, type, and half-life of the therapeutic antibody. This feature offers some control and reversibility lacking in conventional CAR designs, reducing the risk of cytokine release syndrome and other immune-related toxicities.

## Challenges and limitations

The development of FcγR-CR T cells marks a creative and hopeful approach in cancer immunotherapy. This approach offers several benefits over traditional CAR T-cell therapy by targeting TAAs with chimeric antigen receptor (CAR) T cells expressing Fcγ receptors (FcγRs). This includes a universal targeting platform, reduced antigen escape, possible synergistic interactions with other drugs, and enhanced control and safety. Still, several challenges and limitations remain that could compromise the clinical effectiveness of FcγR-CR T cell therapy, despite its promising qualities. These issues relate to the molecular properties of FcγRs, the immune system’s response to altered T cells, and the tumor microenvironment (TME).

Polymorphisms in the FcγR receptors, particularly in the CD16 receptor (FcγRIIIa), constitute a major barrier for clinical translation of FcγR-CR T cells. Activity of FcγR-CR T cells depends on the CD16 receptor, which mediates ADCC. Many genetic variants of CD16, notably the V158F polymorphism, can significantly affect the receptor’s affinity and function [94, 127]. The CD16 V158F variant shows lower affinity for IgG1 antibodies, which could compromise the efficacy of FcγR-CR T cell therapies relying on this receptor for tumor cell destruction [157, 158]. Patients with the CD16 V158F genotype might therefore show a lower therapeutic response, highlighting the need to consider individual polymorphisms in the development of customized FcγR-CR T cell therapies. Variation in receptor function among patients makes it somewhat challenging to standardize treatment protocols and achieve optimal outcomes across different patient populations.

FcγR-CR T cell therapy carries a risk of immunogenicity, much as traditional CAR T cell treatments. Altered receptor expression on T cells can trigger immunological reactions against the changed cells, causing their death and possibly limiting the duration of therapeutic efficacy [159]. Activating these altered T cells inside the tumor microenvironment could trigger cytokine release syndrome (CRS), a severe inflammatory reaction with potentially life-threatening effects [160]. Defined by the release of pro-inflammatory cytokines, particularly interleukin-6 (IL-6), CRS could cause fever, low blood pressure, and organ damage. The use of FcγR-CR T cells might aggravate CRS by simultaneously activating the engineered receptor and the innate immune system via the Fcγ receptor pathway, a known issue in CAR T cell therapy. FcγR-CR T cells may have one benefit: they can adjust antibody dose and half-life to maximize T cell activation, potentially reducing CRS severity. Developing strategies to control cytokine release, including the use of small-molecule inhibitors or antibodies targeting IL-6, could further reduce this issue.

Antibody rivalry is a significant barrier to the effectiveness of FcγR-CR T cell therapies. FcγR-CR T cells function by binding to tumor-associated antigens covered by therapeutic antibodies. In the patient’s circulation, higher levels of natural IgG can disrupt the binding of FcγR-CR T cells to the Fc region of these antibodies, hence reducing the effectiveness of tumor cell targeting [161]. Patients with elevated circulating antibody levels, particularly those who have received prior antibody treatment or have persistent illnesses, find antibody competition particularly difficult. This competition could reduce the ability of FcγR-CR T cells to bind to cancer cells, thereby impairing efficient tumor cell killing and reducing the therapeutic efficacy of the treatment. Higher-affinity antibodies or antibody fragments specifically engineered for FcγR binding could be developed to provide optimal interaction between FcγR-CR T cells and tumor cells, even in the presence of increased endogenous antibody levels, thereby addressing this problem.

The effectiveness of FcγR-CR T cells in cancer therapy remains significantly constrained by the TME. The tumor microenvironment is often characterized by an immunosuppressive setting that could hinder T cell invasion, activation, and activity [162]. The TME in many cancers, substantial tumors, is made up of various components, including immunosuppressive cytokines, regulatory T cells (Tregs), and myeloid-derived suppressor cells (MDSCs), which dampen the immune response and hinder ideal immune cell function [163]. As with previous T cell treatments, FcγR-CR T cells must overcome these immunosuppressive mechanisms to enter the tumor, proliferate, and induce cell death. Furthermore, the effectiveness of antibody treatments may be compromised by the tumor microenvironment, hence reducing the ability of FcγR-CR T cells to find and destroy cancer cells. Approaches that combine FcγR-CR T cell therapy with immune checkpoint inhibitors, which block inhibitory signals in the tumor microenvironment, may enhance the effectiveness of this strategy and support T cell activity.

Though FcγR-CR T cell therapy shows great promise as a complex and powerful tool in cancer immunotherapy, many challenges must be addressed to fully realize its potential. Variations in FcγR polymorphisms hamper the efficient use of this medicine in cancer therapy by increasing the risk of immunogenicity and cytokine release syndrome, rivalry with endogenous antibodies, and an immunosuppressive tumor microenvironment. Dealing with these issues requires continuous study to optimize FcγR-CR T cell design, improve side-effect control, such as CRS, and develop combination treatments to increase effectiveness. Improving the clinical translation of FcγR-CR T cells and ensuring their broad use in treating certain cancers depends on reducing these limitations.

FcγR-CR T-cell therapy has its own distinct advantages and disadvantages. From the clinical safety perspective, it presents unique risks and benefits compared to currently available cell-based immunotherapy options. The major downside of autologous CAR-T product generation is the extremely high association with both high frequency and high severity of cytokine release syndrome (CRS), in part due to the extreme T-cell activation induced by these products. In contrast to autologous CAR-T cells, which produce very specific targeting for a highly localized tumor, while also causing the highest incidence and severity of CRS through the rapidity and duration of T-cell activation and prolonged persistence [164, 165], Allogeneic NK-cell products typically have the benefit of also having a lower likelihood and severity of CRS [166, 167]; however, because they only have a limited period of persistence, the duration of effect against the target would be shorter than for autologous CAR-T cells. FcγR-CR T-cells fall into a transitional area between autologous CAR-T cells and NK-cells in the ability to modulate CD8 + T-cell effector functions on a dose-dependent basis through the antibody-mediated engagement of Fcγ receptors, thus allowing for improved management of treatment-related toxicities [63]; however, it should also be noted that the engagement of Fcγ receptors may augment the activation of downstream components of the cytokine signaling cascade and potentially create competition for binding between the engineered T-cell’s FcγR and other normal immune cells expressing FcγR on their surface, resulting in either reduced efficacy if the T-cell is unable to effectively bind to the antibody or enhanced targeted immune response if the T-cell and/or other immune cells are able to successfully compete for binding to the antibody [168]. In addition to these considerations, multiple other factors may contribute to the degree of immune response observed with engineered T-cell products, including the potential immunogenicity of the engineered receptor constructs and the influence of Fc receptor isotype and/or affinity on both efficacy and system-wide inflammatory responses [59, 165]. Therefore, as the clinical applications of FcγR-CR T-cell platforms continue to develop, the careful selection of antibodies and engineered receptors and the use of dosing strategies that will fully utilize antitumor potency with appropriate levels of safety are essential.

## Future directions for the use of FCγR-CR T cells in cancer therapy

CAR-T Cell therapies have seen incredible results in blood cancers, but treating solid tumors is still difficult because of diverse antigen expression throughout solid tumors, immunosuppressive tumor microenvironments, and safety issues surrounding their use. Adoptive cell therapy faces many challenges in solid tumors, including antigen heterogeneity, dynamic antigen loss, limited immune cell migration into the tumor, and high levels of immunosuppression in the tumor microenvironment. Because of these issues, traditional CAR-T cell therapies have not yet proven successful in treating solid tumors [169, 170]. In addition, the development of CAR-T cells engineered to express FcγR allows for a unique approach to using CAR-T cells, as they aren’t predetermined based solely on their target antigens; they will accept many different types of monoclonal antibodies for targeting, in addition to any existing CAR/TCR that may have already been used in the case of a patient [171]. By using multiple monoclonal antibodies for redirection, the FcγR-CR T-cell can target multiple antigens expressed in the tumor, reduce the effects of antigen escape, and even treat patients with intratumor heterogeneity, especially in cases of patients who develop resistance to multiple therapies simultaneously [171]. Because of the ability to access multiple antigen targets and the ability to control T-cell activation based on the availability and quantity of the antibody, the FcγR-CR T-cell represents a platform with many additional advantages for the treatment of solid tumors that continues to see limitations from safety concerns and other factors associated with CAR-T-cells that have been designed based on fixed specificity [170, 171].

FcγR-CR T cells have been shown in several preclinical studies to be effective, but it remains to be determined how effective these lymphocyte-based products will be within the broader spectrum of cellular immunotherapies. FcγR-CR T cells fill a gap between CAR-T cells made from a patient’s own lymphocytes that are used to treat chronic malignancies and NK cells derived from a donor (allogeneic) for quickly administering and prepackaging as anti-cancer therapies. While CAR-T cells from a patient can survive long enough in a patient to be effective against their malignancy (blood malignancies in particular), CAR-T cells can be challenging to manufacture as they must be manufactured specifically for each patient, have a limited target range, and rapidly lose their killing capacity once they are activated in the patient [169]. Because of their enhanced killing capacity from antibody addition, donor-derived NK cell therapies are available immediately off the shelf and produce virtually no toxicity. On the other hand, these NK cells typically do not survive for long after administration, do not proliferate, and have limited in vivo survival capacity [59, 172]. In contrast to donor-derived NK cell therapies, FcγR-CR T cells can persist indefinitely in patients and develop disease-specific memory. Additionally, FcγR-CR T cells offer an intermediate form of cytotoxic anti-tumor therapy, combining the antibody-targeting capabilities of NK cells with the potential for durability and flexibility. Thus, this combination of features is expected to improve therapeutic responses to patient needs in terms of safety and efficacy, and to provide a platform for modulating T-cell cytotoxicity by administering antibodies that target heterogeneity in solid tumors.

Using FcγR-CR T cells in cancer therapy marks a promising development in immuno-oncology. Many important developments—including affinity-engineered FcγRs, bispecific antibodies, allogeneic universal T cells, regulatable expression systems, and combination therapies—will determine the future of FcγR-CR T cell treatment in cancer. These methods could significantly increase the specificity, safety, and effectiveness of FcγR-CR T cells. 

### Comparative positioning of FcγR-CR T cells among cellular immunotherapies

FcγR-CR T cells represent a unique subclass of CAR T cells. They lie at the intersection of both autologous (self) CAR-T-cells and allogeneic (donor) NK-cells, integrating the persistent adaptive properties of T-cells with the antibody-mediated targeting properties typically associated with non-adaptive immune effectors such as NK-cells. FcγR-CR T-cells will differ from traditional CAR-T in that they utilize clinically relevant monoclonal antibodies (mAbs), allowing a modular approach to multi-antigen targeting and externally controlled activation of effector T-cells upon mAb binding to target cells. In contrast, under normal circumstances, NK cells provide a rapid and efficient means of destroying target cells in the body. FcγR-CR T-cells will provide this same speed and efficiency, with additional characteristics associated with the persistence and expansion of T-cells after being given the correct signal. Moreover, because of the hybrid nature of FcγR-CR T cells, they will face distinct translational and clinical development challenges. All of these factors must be balanced with the effector T cells’ ability to remain active while still recognizing additional targets in the body.

### Affinity-engineered FcγRs to enhance tumor targeting

The variability in the affinity of FcγRs for therapeutic antibodies is a fundamental issue in FcγR-CR T cell therapy. Specifically, affinity engineering of Fcγ receptors, notably CD16 (FcγRIIIa), could significantly improve targeting of cancer cells. Enhanced affinity FcγRs can bind antibodies that opsonize cancer cells more efficiently, thereby increasing CAR T cells’ tumor-eradicating capacity. Especially in cases of low or variable antigen expression, high-affinity variants of FcγRs could increase the likelihood that FcγR-CR T cells interact with tumor cells [157, 161]. This might improve the targeting of cancer cells that could otherwise escape immune detection. Moreover, creating FcγRs with improved binding affinity should help overcome antigenic modulation or escape variants, thereby allowing FcγR-CR T cells to effectively target cancers as they evolve over time.

### Bispecific antibodies or Fc-engineered mAbs to increase specificity and reduce off-tumor toxicity

Using bispecific antibodies or Fc-engineered mAbs offers a feasible way to improve FcγR-CR T cell therapy. Engineered to bind to two separate antigens simultaneously, bispecific antibodies could improve the specificity of FcγR-CR T cells for cancer cells and lower off-tumor damage. For instance, bispecific antibodies that target both a tumor-specific antigen and the Fc region of antibodies could ensure that FcγR-CR T cells only interact with tumor cells, especially targeted by the antibody [173]. Targeting healthy cells unrelated to the tumorigenic process with FcγR-CR T cells may increase non-specific binding and off-tumor harm, which this approach could help to reduce. Furthermore, altering the Fc region of monoclonal antibodies to enhance their affinity for Fcγ receptors could further fine-tune treatment by ensuring that FcγR-CR T cells are triggered only in specific TAAs.

### Allogeneic universal FcγR-CR T cells for off-the-shelf therapies

One significant disadvantage of current CAR T cell treatments is the need for autologous T cell isolation, which is both expensive and time-consuming. Developing allogeneic universal FcγR-CR T cells for off-the-shelf therapy offers a promising path forward. Allogeneic T cells would remove the need to collect the patient’s T cells, speeding treatment and reducing logistical issues. Using allogeneic T cells in clinical settings poses the main challenge of the risk of graft-versus-host disease (GVHD), in which the given T cells attack the patient’s tissues. Researchers are looking at genetically modifying T cells to prevent immunological rejection. One approach is to remove genes encoding major histocompatibility complex (MHC) molecules, which are essential for initiating immune responses. Designed to be injected into any patient without causing GVHD, universal donor T cells could provide allogeneic FcγR-CR T cells, offering a standardized treatment approach for cancer patients and enabling faster availability than autologous treatments.

### Regulatable expression systems for safety and reversibility

Given that their uncontrolled activation could have significant negative consequences, such as cytokine release syndrome (CRS), ensuring the safety of FcγR-CR T cells is vital. A possible way to address this issue is by creating CAR T cells with regulatable expression systems. These technologies enable CAR T cells to be temporarily activated or deactivated in response to outside influences. For example, chemical inducers or small-molecule switches can control the expression of FcγR-CR, allowing the therapy to be modified as needed [159]. Rapid cessation of CAR T cell activation may be particularly useful for reducing adverse effects such as CRS. Furthermore, suicide genes could be included in FcγR-CR T cells to enable their controlled destruction in case of notable negative consequences. The development of these safety switches would improve control, strengthening the overall safety profile of FcγR-CR T cell therapies and enabling their widespread use.

The selection of an antibody isotype can significantly impact the effectiveness and safety of an Fc-dependent immunotherapy. Antibodies bind to and activate different types of Fcγ receptors with varying degrees of affinity, which determines what effector function is activated downstream of that receptor interaction. The four subclasses of human IgG have very different affinities for activating and inhibiting FcγRs as well as for complement component C1q; therefore, they produce very different effector outcomes. IgG1 has a high affinity for activating FcγRs (such as FcγRIIIa), and has strong binding to C1q, which means that IgG1 can mediate high levels of antibody-dependent cellular cytotoxicity (ADCC), antibody-dependent cellular phagocytosis (ADCP), and complement-dependent cytotoxicity (CDC). Therefore, when developing antibodies targeting tumors that require strong activation of effector functions, IgG1 is the preferred isotype. In contrast, IgG2 and IgG4 bind to most of the activating FcγRs with very low affinity and are unable to activate CDC; therefore, these isotypes may produce less effector cell recruitment and cytotoxicity than IgG1. The choices made between antibody isotypes can have major impacts on the performance and the safety of an FcγR-based immunotherapy due to the fact that the various subclasses of human IgGs bind differently to different Fc gamma receptors as well as complement components, ultimately resulting in different effector functions and varying degrees of potential inflammatory effect [71, 174]. Activation through the activating FcγRs such as FcγRI, FcγRIIa and FcγRIIIa is strongest when using IgG1. Along with its superior ability to bind C1q (the first component of the complement system), IgG1 is responsible for the most powerful ADCC, ADCP, and CDC, and has become the optimal isotype for antibodies directed against tumors intended to produce robust effector activity [59, 67, 175]. Conversely, IgG2 and IgG4 have very weak binding to the majority of activating FcγRs and a diminished ability to activate complement; therefore, they lead to a more dampened effector engagement and greater attenuation of the inflammatory signal [67, 175]. The comparatively low ratios of receptor engagement for IgG2 and IgG4 also make these antibodies very attractive in circumstances such as immune checkpoint blockade, where effector functions mediated through Fc are not needed or are potentially toxic when combined with the blockade of the immune checkpoint, leading to a much lower risk for inflammatory side effects and systemic toxicity [28]. Although IgG1 has a superior ability to activate complement, potentially increasing antitumor receptor-mediated cytotoxicity, this heightened activation may also increase the risk of off-target complement activation and damage to surrounding tissues [176, 177]. The isotypes have specific effects modulated by single nucleotide polymorphisms or mutations in FcγRs and through the environmental factors associated with the tumor microenvironment that impact both expression and activation thresholds of FcγRs, thus, demonstrating the value of careful selection of antibody isotype and the rational engineering of Fcs in FcγR-CR T cell therapeutic design to achieve the maximum antitumor activity while maintaining a protective safety profile [25, 178].

### Combination with checkpoint inhibitors, cytokine support, or metabolic modulators to overcome TME resistance

Various cancer treatments, notably FcγR-CR T cells, find the TME a significant barrier to their effectiveness. The tumor microenvironment is often characterized by immune suppression, which may hinder T cell infiltration, activation, and function. Combining FcγR-CR T cell therapy with checkpoint inhibitors, such as PD-1/PD-L1 inhibitors, which can enhance T cell activation and restore immune function in the tumor microenvironment, offers a viable approach to overcoming this challenge. Cytokine support—especially interleukins like IL-2 or IL-15—may improve the development and persistence of FcγR-CR T cells at the tumor site, hence increasing their efficacy [179]. Moreover, metabolic regulators that alter the energy dynamics of the tumor microenvironment could help create a more T cell-permissive environment, thereby improving the effectiveness of FcγR-CR T cells. Blocking immunosuppressive metabolites, such as lactate or tryptophan, could help overcome the metabolic barrier in the tumor microenvironment, thereby increasing the effectiveness of FcγR-CR T cells.

With many innovative ideas expected to improve its efficacy, safety, and accessibility, the outlook for FcγR-CR T cell treatment is encouraging. To fully fulfill the potential of FcγR-CR T cell therapy, it is essential to enhance tumor targeting through the improvement of FcγRs, the application of bispecific antibodies or Fc-engineered monoclonal antibodies to minimize off-tumor toxicity, the development of universal allogeneic T cells for more accessible treatment options, and the incorporation of regulatable expression systems to ensure greater safety. Moreover, combining FcγR-CR T cells with checkpoint inhibitors, cytokine support, and metabolic modulators offers a powerful way to overcome the immunosuppressive tumor microenvironment and enhance therapeutic effectiveness. These advances could enable highly efficient, less harmful cancer treatments, thereby improving patient outcomes and extending the use of FcγR-CR T cell therapy in cancer.

## Conclusion

Particularly for solid tumors, which pose significant challenges due to their heterogeneity, immune evasion, and unfavorable tumor microenvironment (TME), the emergence of FcγR-driven chimeric receptor (CR) T cells represents a promising and flexible approach in cancer immunotherapy. These altered T cells can be guided by monoclonal antibodies (mAbs) toward a varied spectrum of tumor-associated antigens (TAAs) by replacing the conventional single-chain variable fragment (scFv) with the extracellular domain of Fcγ receptors—such as CD16a, CD32a, or CD64a—thereby enabling antigen-agnostic targeting and rapid control of T cell activation. This review article highlights the many advantages of FcγR-CR T cells over traditional CAR T platforms, including flexibility, reversibility, and the ability to work in concert with existing monoclonal antibody treatments. Now, future directions are focused on three vital innovations: Future directions are now concentrated on three critical innovations: [1] The development of affinity-engineered FcγRs to maximize tumor targeting while minimizing off-target effects; [2] the amalgamation with bispecific antibodies or Fc-engineered monoclonal antibodies to augment selectivity and diminish systemic toxicity; [3] the development of allogeneic, universal FcγR-CR T cells for scalable, ready-to-use applications; [4] the integration of regulatable expression systems to improve safety profiles; and [5] combinatorial approaches with checkpoint inhibitors, cytokine support, or metabolic modulators to surmount resistance mechanisms within the tumor microenvironment. FcγR-CR T cells demonstrate the convergence of adaptability, specificity, and immunological precision as antibody engineering advances and our understanding of the tumor microenvironment deepens. Especially for resistant and metastatic solid tumors, they are an advanced platform that could transform antibody-guided adoptive cell therapy. Fulfilling their therapeutic potential and integrating them into the arsenal of tailored cancer treatments will depend on ongoing preclinical development and thorough clinical validation.

## Data Availability

No datasets were generated or analysed during the current study.
